# Targeting imidazole-glycerol phosphate dehydratase in plants: novel approach for structural and functional studies, and inhibitor blueprinting

**DOI:** 10.3389/fpls.2024.1343980

**Published:** 2024-03-15

**Authors:** Wojciech Witek, Joanna Sliwiak, Michal Rawski, Milosz Ruszkowski

**Affiliations:** ^1^ Department of Structural Biology of Eukaryotes, Institute of Bioorganic Chemistry, Polish Academy of Sciences, Poznan, Poland; ^2^ Cryo-EM Facility, SOLARIS National Synchrotron Radiation Centre, Krakow, Poland

**Keywords:** IGPD, HISN5, herbicide design, enzyme kinetics, histidine biosynthesis

## Abstract

The histidine biosynthetic pathway (HBP) is targeted for herbicide design with preliminary success only regarding imidazole-glycerol phosphate dehydratase (IGPD, EC 4.2.1.19), or HISN5, as referred to in plants. HISN5 catalyzes the sixth step of the HBP, in which imidazole-glycerol phosphate (IGP) is dehydrated to imidazole-acetol phosphate. In this work, we present high-resolution cryoEM and crystal structures of *Medicago truncatula* HISN5 (*Mt*HISN5) in complexes with an inactive IGP diastereoisomer and with various other ligands. *Mt*HISN5 can serve as a new model for plant HISN5 structural studies, as it enables resolving protein-ligand interactions at high (2.2 Å) resolution using cryoEM. We identified ligand-binding hotspots and characterized the features of plant HISN5 enzymes in the context of the HISN5-targeted inhibitor design. Virtual screening performed against millions of small molecules not only revealed candidate molecules but also identified linkers for fragments that were experimentally confirmed to bind. Based on experimental and computational approaches, this study provides guidelines for designing symmetric HISN5 inhibitors that can reach two neighboring active sites. Finally, we conducted analyses of sequence similarity networks revealing that plant HISN5 enzymes derive from cyanobacteria. We also adopted a new approach to measure *Mt*HISN5 enzymatic activity using isothermal titration calorimetry and enzymatically synthesized IGP.

## Introduction

Since the 1960s, more than 250 weed species have become resistant to over 150 herbicides, mostly because of repeated use (Gould et al., 2018; Beckie et al., 2021; Gaines et al., 2021). Current herbicides also raise safety concerns and have negative impacts on the environment. Recent reports have delivered evidence that the most common herbicide, glyphosate, is harmful to honeybee broods, impairing their sensory and cognitive abilities and gut microbiome (Motta et al., 2018; Farina et al., 2019; Vázquez et al., 2020). Other herbicides, such as triazines (atrazine, hexazinone), anilides (acetochlor, alachlor), and carbamates, enter aquatic environments and accumulate in coral reefs (Tyohemba et al., 2022). They cause acute toxicity, leading to reduced zooxanthellar photosynthetic efficiency (Negri et al., 2005), resulting in bleaching, reduced reproductive output, and partial or full-colony mortality (Cantin et al., 2007). These factors incite the development of new herbicides to ensure that eight billion people on the planet can be fed sustainably. In this view, the histidine biosynthetic pathway (HBP) has become a promising new target for the development of herbicides (Hall et al., 2020).

The HBP occurs in bacteria, archaea, plants, and other lower eukaryotes, such as yeast or protozoans, but is absent in animals. It has been intensively studied since the 1950s (Miller and Bale, 1952; Adams, 1954; Ames and Mitchell, 1955; Ames, 1957a, Ames, 1957b), mostly in *Escherichia coli* and *Salmonella typhimurium*. The research was later continued by Ames, Brenner, and Martin, who identified all enzymes, metabolic intermediates, and by-products (Brenner and Ames, 1971; Martin et al., 1971). Studies of the HBP in plants started later due to the lack of auxotrophic mutants and the complicated biochemistry behind the pathway (Wiater et al., 1971c; Ingle, 2011). In fact, the plant HBP was genetically deciphered in 2010, as the last amino acid biosynthetic pathway (Petersen et al., 2010).

The overall organization of the HBP is conserved across kingdoms, but there are significant differences between homologous enzymes. These differences were caused by genetic events during evolution, such as gene duplications, elongations, horizontal gene transfers (HGT), and gene fusions resulting in the emergence of bi- or even trifunctional enzymes (Brilli and Fani, 2004; Stepansky and Leustek, 2006; Reyes-Prieto and Moustafa, 2012; Del Duca et al., 2020; Rutkiewicz et al., 2023). The HBP consists of ten steps (eleven reactions considering the glutaminase activity of HISN4 auxiliary), catalyzed in plants by eight enzymes that are named HISN1-8 by their action in the HBP sequence. Each of the eight enzymes in plants is encoded by nuclear DNA and contains an N-terminal chloroplast transit peptide (Fujimori and Ohta, 1998).

**Figure 1:**
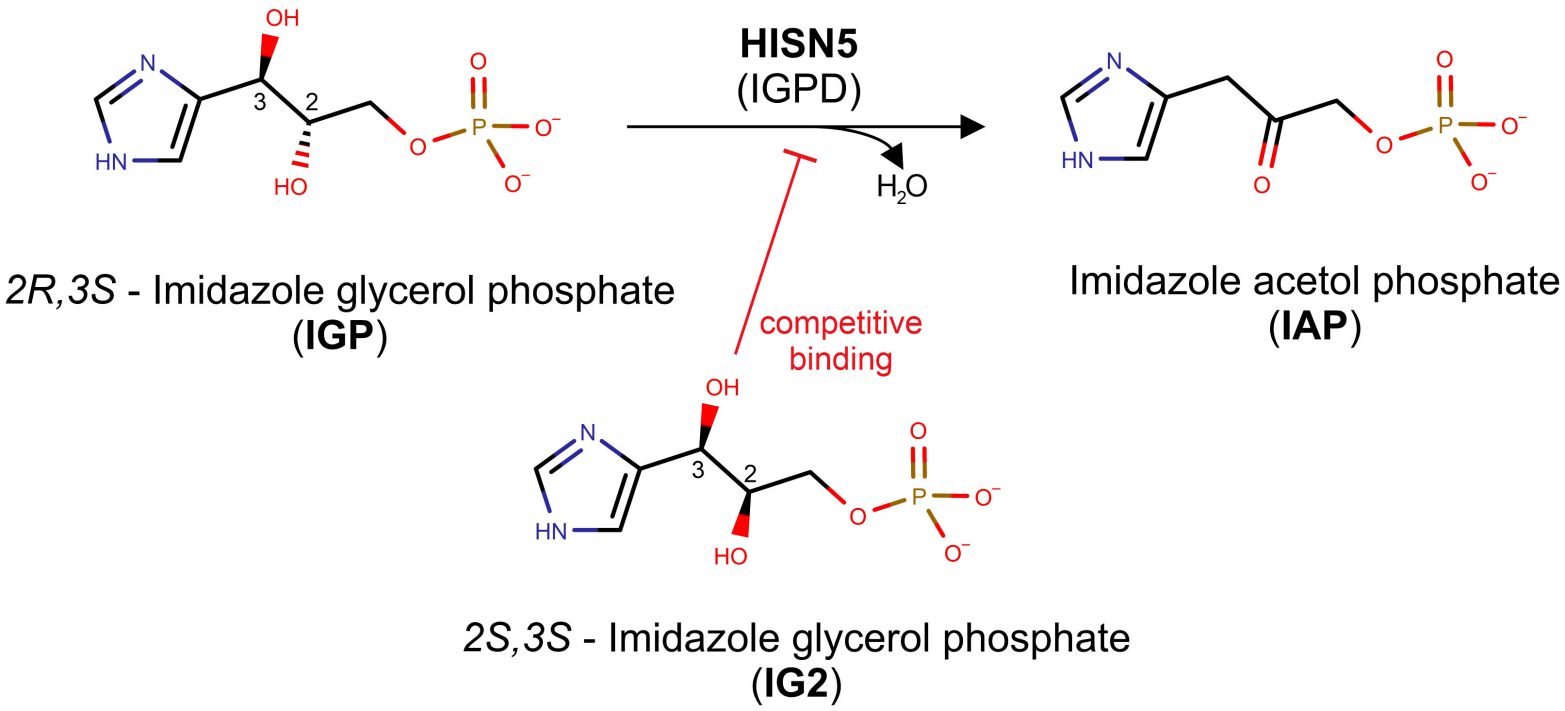
Scheme of the reaction catalyzed by HISN5/IGPD. The top reaction depicts how the *Mt*HISN5 substrate, *2R,3S* imidazole-glycerol phosphate (IGP), is dehydrated to imidazole-acetol phosphate (IAP). The *2S,3S* diastereoisomer of IGP (IG2), which binds competitively to IGP, is shown in the bottom.

This work focuses on D-*erythro*-imidazole-glycerol phosphate dehydratase (IGPD, EC 4.2.1.19) or HISN5 as it is referred to in plants. Notably, IGPD-encoding genes are named inconsistently between kingdoms, for example, *HISN5* in plants, *HIS3* in yeast, or *HisB* in bacteria (Muralla et al., 2007). Interestingly, in most species, IGPDs are monofunctional enzymes; however, in some bacterial phyla, they perform two HBP reactions as a result of gene fusion between genes encoding IGPD and histidinol-phosphate phosphatase (HPP, EC 3.1.3.15), which are HISN5 and HISN7 counterparts in plants (Brilli and Fani, 2004). Plant HISN5 catalyzes the sixth step of the HBP, in which imidazole-glycerol phosphate (IGP) is dehydrated to form imidazole-acetol phosphate (IAP, **Figure 1**). HISN5 activity in plants was first described in 1971 (Wiater et al., 1971c) but the first plant enzyme was purified from wheat germ in 1993 (Mano et al., 1993). HISN5 has been considered for about fifty years as a potential target for triazole compounds, e.g., amitrole, 3-amino-1,2,4-triazole, and 2-hydroxy-3-(1,2,4-triazol-1-yl) (C348) (Hilton et al., 1965; Wiater et al., 1971a, Wiater et al., 1971b; Kishore and Shah, 1988; Rawson et al., 2018). However, amitrole is a non-selective herbicide with off-target effects. As reported by Furukawa et al., amitrole shows carcinogenic activity in rats, mice, and humans (Furukawa et al., 2010). Therefore, there is great demand for more selective HISN5 inhibitors that exhibit fewer side effects.

*Arabidopsis thaliana* was the first model for structural studies of plant HISN5 and provided the groundwork for structure-based inhibitor design. *At*HISN5 occurs in two isoforms, A and B, which are 270 and 272 amino acid residues long, respectively. Its fold resembles a sandwich constituted of a bundle of four, centrally located, α-helices which are surrounded by two β-sheets from both sides. This metalloenzyme utilizes Mn^2+^ for proper folding and catalysis (Glynn et al., 2005b). The plant HISN5, with its 24-meric structure, could potentially offer a plethora of druggable sites, not only in the active site but also at unique channels, clefts, and inter-subunit interfaces. Such hot-spots often make a major contribution to the protein-ligand binding free energy gain and are therefore an important factor in the discovery of bioactive compounds (Zerbe et al., 2012). The first crystal structure of *At*HISN5, at a 3 Å resolution, was obtained in 2005, providing insights into manganese cations coordination and reaction mechanism (Glynn et al., 2005b). A decade later, a series of *At*HISN5 crystal structures at highly improved resolution (1.1 – 1.5 Å) offered the first details about substrate and inhibitor positioning (Bisson et al., 2015, Bisson et al., 2016). The advent of cryogenic electron microscopy (cryoEM) brought about the first microscopic structure of a complex of *At*HISN5 with a triazole inhibitor, obtained at 3.1 Å (Rawson et al., 2018). At that resolution, it was only possible to confirm the presence of the inhibitor in the EM map without providing details about its binding mode. However, rational herbicide design requires high-quality structures of enzymes and an understanding of the interactions that occur at the molecular level. Hence, there is a need for a better model of the plant HISN5 enzyme to study its interactions with small molecules using cryoEM.

The HISN5 enzyme from a model legume, *Medicago truncatula*, was selected for this study for various reasons. Structural studies require a prior preparation of expression constructs, e.g., cleavage of signal peptides, testing different ranges, etc. The availability of *M. truncatula* genomic sequence makes such modifications feasible (Young et al., 2011; Burks et al., 2018). Furthermore, this study is a continuation of work to provide a complete picture of the HBP in legumes. So far, we published structures of HISN1 (Ruszkowski, 2018), HISN2 (Witek et al., 2021), HISN6 (Rutkiewicz et al., 2023), HISN7 (Ruszkowski and Dauter, 2016), and HISN8 (Ruszkowski and Dauter, 2017). The model *M. truncatula* is closely related to *Medicago sativa* (lucerne or alfalfa), an economically and environmentally important forage crop (Mueller-Harvey et al., 2019; Hrbácková et al., 2020; Sakiroglu and Ilhan, 2021).

Although there are available *At*HISN5 structures, our research was motivated by the lack of high-resolution cryoEM structures of plant HISN5 enzymes, which could allow to study protein interactions with small molecules. Therefore, we established a pipeline for HISN5 cryoEM research that yields maps at a resolution allowing to study interactions with ligands. Our experimental results were combined with computational approach to describe ligand binding hot-spots and potential pharmacophores for future design of novel inhibitors. So far, only a few molecules have been the subject of interest in terms of plant HISN5 inhibition. The similarity of plant, fungal, and bacterial IGPD enzymes in the active site poses a potential threat for off-target (antimicrobial) activities introduced by HISN5-targeted herbicides. To provide a background for reaching selectivity at the kingdom level, we analyzed and compared residue conservation for plants and other organisms to identify a region near the active site which is specific to plants. Finally, we developed a new approach to measure catalytic properties of *Mt*HISN5. This was motivated by two factors, i.e., poor availability of IGP on the market and its contamination with an inactive diastereoisomer (Saika et al., 1993; Bisson et al., 2015), able to bind competitively instead of the *bona fide* substrate.

## Results and discussion

### CryoEM and crystal structures – an overview of *Mt*HISN5 structural features

**Figure 2:**
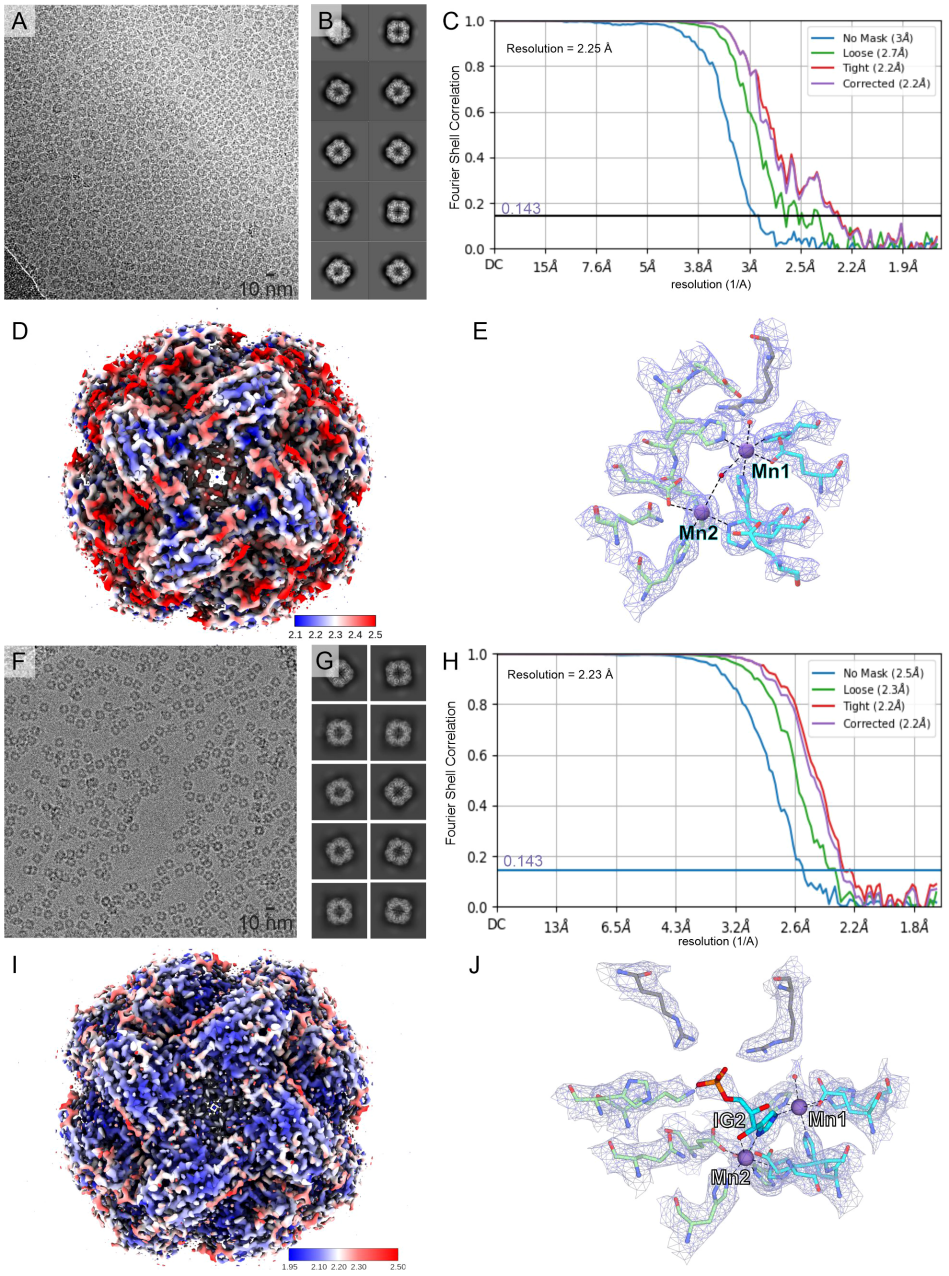
CryoEM structures of *Mt*HISN5. **(A–E)** present the *Mt*HISN5-unliganded structure, whereas **(F–J)** refer to the *Mt*HISN5-IG2 complex; **(A, F)** representative micrographs; **(B, G)** example 2D classes; **(C, H)** Fourier-shell correlation curves; **(D, I)** maps colored by local resolution; and **(E, J)** map fragments within the active site.

This work describes results obtained from five experimental structures of *Mt*HISN5, including three crystal structures at 1.55, 1.69, and 2.2 Å resolutions (**Supplementary Figures S1A–C**) and two cryoEM structures resolved at 2.4 Å (*Mt*HISN5-unliganded, PDB ID: 7OJ5) and 2.2 Å (complex *Mt*HISN5 with *2S,3S*-IGP (referred to as IG2), PDB ID: 8QAV) (**Figure 2**). The latter was obtained using the commercially available IGP (cIGP).

The crystal structure at 1.55 Å was obtained in space group *R*3 and comprises eight chains in the asymmetric unit (ASU). The crystal structure at 2.2 Å is isomorphous to the 1.55 Å structure but contains different ligands, e.g., citrate (CIT, **Supplementary Figure S1D**, see below). We also obtained the structure in space group *I*4 at 1.69 Å resolution with six chains in the ASU. The crystal structures cover the *Mt*HISN5 sequence starting from Gly77 or Ala78 through Arg260/261 (chain and structure-dependent). Both cryoEM structures (unliganded and IG2 complex) have been reconstructed from EM maps with octahedral symmetry, and therefore contain 24 identical protein chains, spanning from Ala78 to Arg261 (IG2 complex) and Arg262 (unliganded).

**Figure 3:**
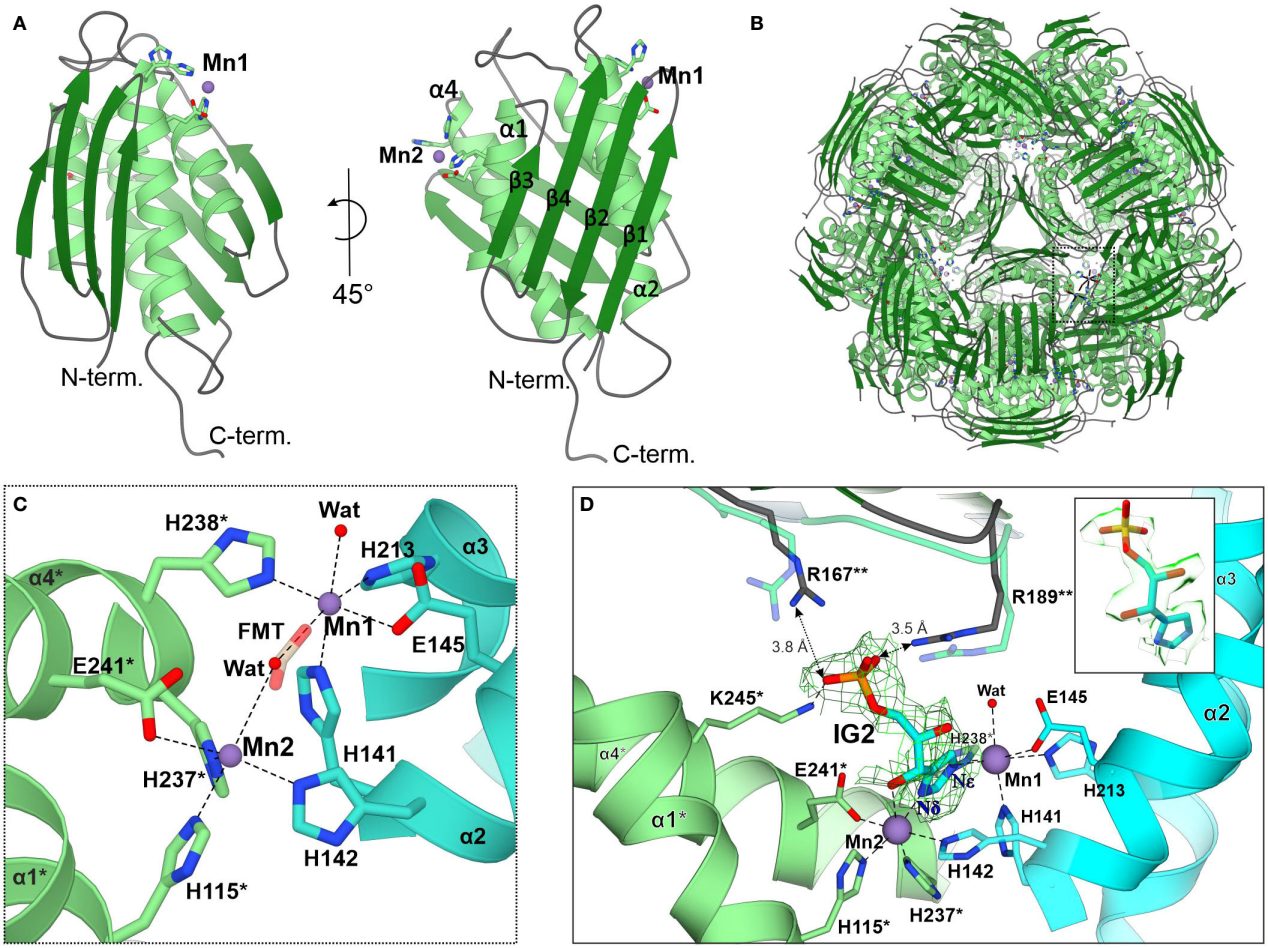
The *Mt*HISN5 structure. **(A)** The subunit of *Mt*HISN5 in two orientations: α-helices are in light green, β-strands are in forest green, and loops are in cyan. Four α-helices form a core bundle surrounded by four β-strands on each side. The N-terminal β-sheet forms an external surface and the C-terminal β-sheet forms the internal surface of the oligomer. **(B)** *Mt*HISN5 24-mer, shown along the three-fold axis. The black, dashed rectangle indicates the regions shown in **(C)**. **(C)** Octahedral coordination of the manganese cations constituting the active site. Formate (FMT, semitransparent) was found in the active sites of crystal structures. Chains are colored differently (light green and turquoise) to emphasize their contribution to the active site formation. The red balls represent the water molecules. Asterisks (*) indicate residues from the counterpart molecule. **(D)** The architecture of the active site in the cryoEM *Mt*HISN5-IG2 complex. The complete substrate binding requires residues from the third (**) subunit. The imidazole ring of IG2 is bound between Mn1 and Mn2 and its phosphate group is held by side chain guanidines of R167** and R189**; semitransparent R167** and R189** present conformation with no ligand in the active site. The cryoEM potential map is contoured 2 Å around IG2 (σ = 0.26) and colored in green. The inset shows the map in semitransparent surface representation, clearly revealing the *2S,3S* configuration in IG2.

The *Mt*HISN5 subunit contains a four α-helix bundle sandwiched between two β-sheets whose four strands are almost perpendicular to each other (**Figure 3A**). In solution, *Mt*HISN5 forms a homo 24-mer with octahedral (432) symmetry (**Figures 2A, F**, **3B**) of approx. 110 Å diameter and a total mass of 540 kDa. It remains unclear why natural selection has promoted 24-merization for IGPDs/HISN5s. Possible drivers may be (i) minimization of energy cost and amino acid usage (Akashi and Gojobori, 2002; Seligmann, 2003), (ii) proteome stability and efficiency of translation (Kepp, 2020), and (iii) cost of gene expression (Frumkin et al., 2017). Another evolutionary driver of oligomerization may be the benefit of cooperative regulation of the enzyme activity. However, to the best of our knowledge, such a property has never been reported for any IGPD enzyme. The dimensions of the *Mt*HISN5 oligomer did not change significantly after the binding of IG2, indicating a lack of major conformational rearrangements. The active site of *Mt*HISN5 (see below) contains two Mn^2+^ cations (Mn1 and Mn2, **Figure 3C**) bound by residues belonging to different subunits. In other species, it has been shown that withdrawal of Mn^2+^ causes HISN5 dissociation to inactive trimers while re-addition of Mn^2+^ or other divalent metal cations (e.g., Co^2+^, Cd^2+^, Ni^2+^, Fe^2+^, and Zn^2+^) reassembles the enzyme (Sinha et al., 2004; Glynn et al., 2005b). However, we did not observe *Mt*HISN5 trimers at any stage of the purification process, even when 40 mM EDTA was used (not shown).

### Characteristics of the *Mt*HISN5 active site

As mentioned above, each subunit of *Mt*HISN5 contains two Mn^2+^ cations (Mn1 and Mn2). The cryoEM and crystal structures share the same pattern of Mn^2+^ coordination (**Figures 3C, D**). Mn1 is coordinated octahedrally by Nϵ of His141, Nϵ of His213, carboxyl of Glu145, Nϵ of His238* from a neighboring subunit (*), and a water molecule. The Mn2 cations are complexed by Nϵ of His142, Nϵ of His115*, Nϵ of His237*, carboxyl of Glu241*, and a water molecule. The Mn2 coordination sphere is incomplete in the *Mt*HISN5-unliganded cryoEM structure, owing to the lower map resolution. The crystal structures were obtained in the presence of formate (FMT) between Mn1 and Mn2, which completes their coordination spheres (**Figure 3C**). The corresponding position is occupied by a water molecule in the unliganded cryoEM structure (**Figure 2E**) or by the imidazole moiety in the *Mt*HISN5-IG2 complex (**Figure 2J**).

The cryoEM *Mt*HISN5-IG2 complex structure reveals detailed information about the most likely substrate positioning before catalysis. The imidazole ring is trapped between manganese ions, facing its Nδ towards Mn2 and Nϵ towards Mn1. While the formation of the active site with the bi-Mn^2+^ cluster requires a contribution of residues from two subunits, a third subunit (**) participates in substrate/product binding by contributing guanidines of Arg167** and Arg189** that bind the phosphate group (**Figure 3D**). However, based on our complex with IG2, these polar H-bonds are rather weak, with the donor-acceptor distances of 3.5 – 3.8 Å. Participation of the corresponding Arg121 of *Mycobacterium tuberculosis* HisB in closing the active site has been pointed out recently by Kumar and coworkers (Kumar et al., 2022).

It must be emphasized that our *Mt*HISN5-IG2 complex structure is a spectacular example where the cryoEM maps are of such a high quality that they permit resolving stereoisomers of ligands bound to a protein. In fact, we expected the reaction product, IAP as the enzyme was incubated with cIGP (at 2 mM concentration) for 2 days prior to the cryoEM grid preparation. We clearly recognized IG2 based on EM maps (**Figure 3D**, inset). The improvement in map resolution is an important advancement compared to *At*HISN5, which yielded a 3.1-Å EM map (PDB ID: 6EZJ), making it difficult to determine the positioning of ligands and water molecules as well as distinguishing between the *R*- and *S*-isomers (IGP vs IG2) (Rawson et al., 2018). In this context, using *Mt*HISN5 as a model and our pipeline for the cryoEM structure-based development of novel herbicides will be a significant improvement.

### *Mt*HISN5 possesses a variety of ligand-binding hot-spots

**Figure 4:**
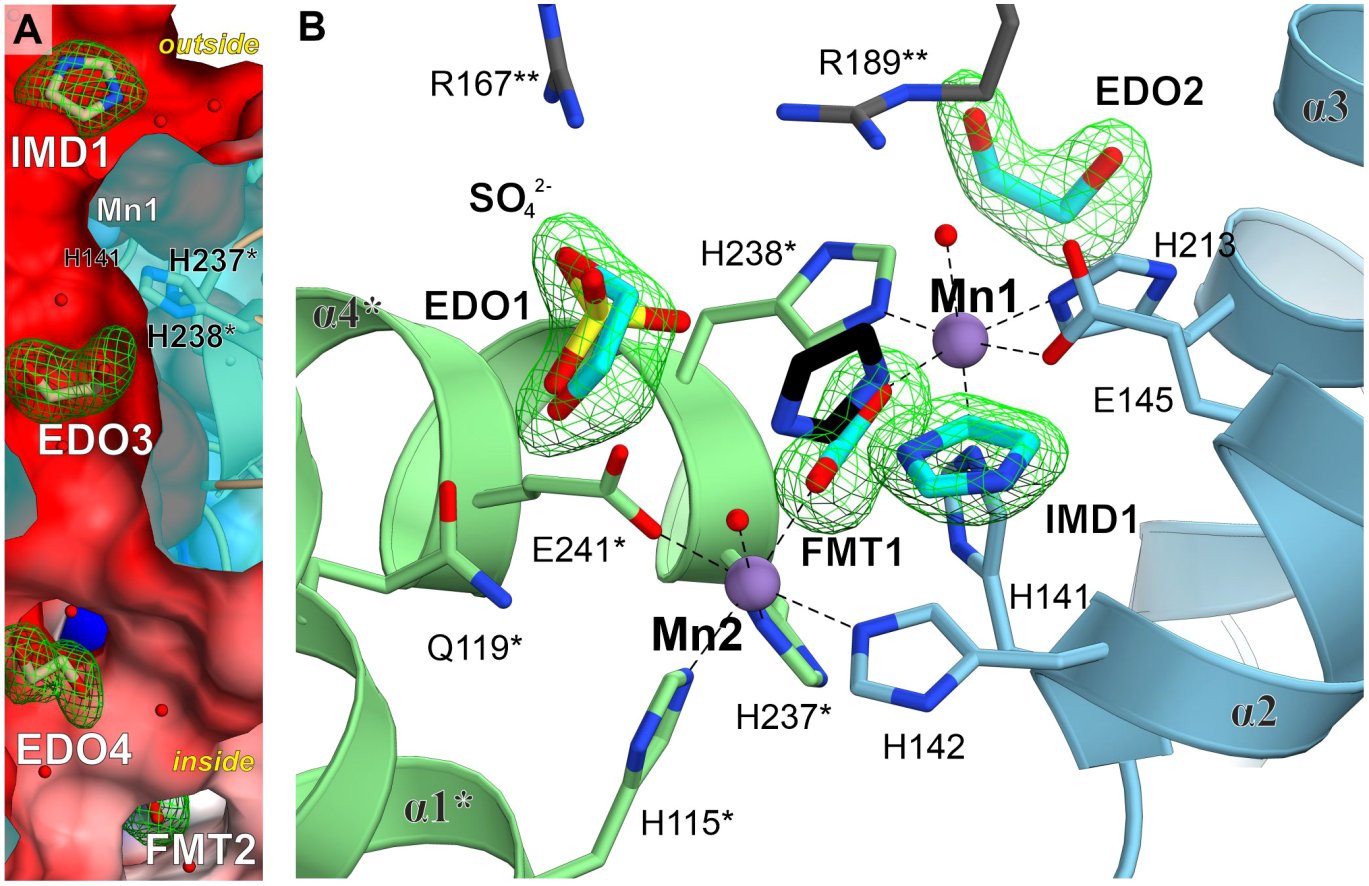
Ligand-binding hot-spots in and near the *Mt*HISN5 active site. **(A)** A molecular tunnel along a two-fold axis with ligands trapped inside. Mn1 is located ~6.9 Å from EDO3 which is in the middle of the tunnel. Polder maps around the ligands are contoured at the 6.8 σ level for IMD, 5.6 for EDO3, 5.0 for EDO4 and 7.3 for FMT2. The *outside/inside* labels indicate protein surfaces. The clipped surface is semitransparent. **(B)** Comparison of small-molecule binding positions. IMD1 in *Mt*HISN5 binds to a different site than in the *At*HISN5 structure (black, PDB ID: 4MU1). Polder maps are contoured at 6.7-10.2 σ levels for this work structures.

In addition to the IG2 observed in our cryoEM complex, we identified several types of molecules bound to *Mt*HISN5, suggesting hot-spots prone to bind certain chemical moieties. The crystal structure at 1.55 Å resolution contains imidazole (IMD), sodium ion, chloride ion, formate (FMT), 1,2-ethanediol (EDO), glycerol (GOL), and tris(hydroxymethyl) aminomethane (TRS). The structure at 1.69 contains FMT, GOL, and TRS. The structure at 2.2 Å contains a chloride ion, FMT, EDO, acetate (ACT), sulfate ion, and CIT. The most abundant binders among these structures are EDOs (n = 41) and FMTs (n = 49). EDOs bind mostly to the inner surface and interfaces between subunits (**Figure 4A**), but a few are also found on the outer surface and in the vicinity of the active site (4-6 Å, **Figure 4B**). FMTs primary location was in the active sites, between Mn1 and Mn2 (**Figures 3C**, **4B**). However, it was very interesting to find that IMD (IMD1, **Figure 4B**) bound not between the Mn^2+^ ions in the active site, but instead was positioned approximately 3.8 Å from the C atom of FMT. This is in contrast to other reported structures (*At*HISN5, PDB ID: 4MU1 (Bisson et al., 2015)), where imidazole mimicked part of the substrate/product (between Mn1 and Mn2). In *Mt*HISN5, IMD1 forms hydrogen bonds with FMT and water. We postulate that the carboxylate and imidazole binding sites can be used as pharmacophores for the design of selective inhibitors of plant HISN5. The fact that we see FMT between Mn^2+^ and imidazole positioned differently suggests that the bi-Mn^2+^ cluster has a high affinity for carboxylate, in addition to the imidazole of IGP or IAP.

**Figure 5:**
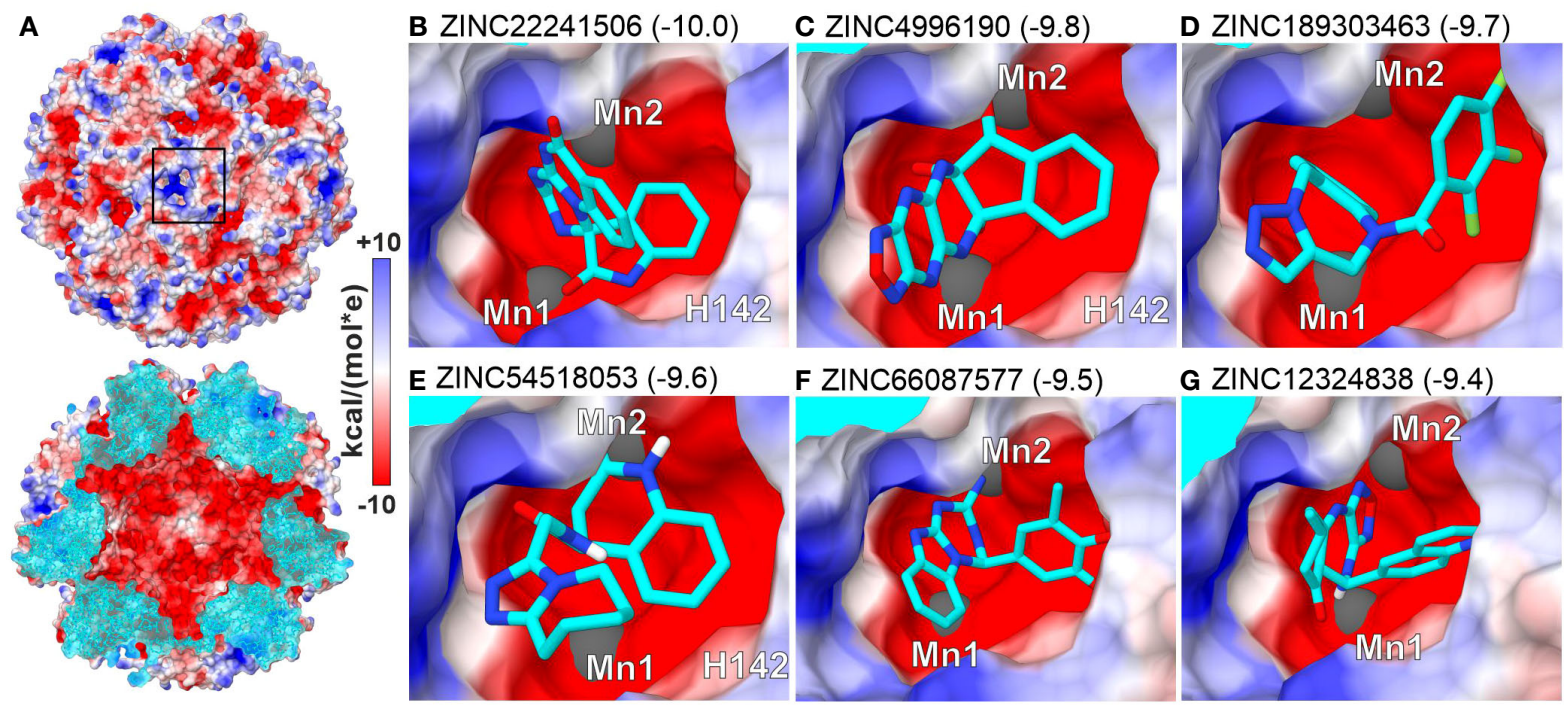
Top-scoring results of virtual screening (VS) at the active site. **(A)** *Mt*HISN5 24-mer surface potential (Coulombic); the cross-section (bottom) of the oligomer reveals a negatively charged inner surface. **(B–G)** (same surface color scheme) show molecules with the highest calculated binding energy gain (kcal/mol).

To search for small molecules representing a broader chemical space, we performed virtual screening (VS) by *in silico* docking 3.3 mln lead-like molecules from the ZINC database (Sterling and Irwin, 2015) in the neighborhood of the *M*tHISN5 active site. Six molecules scoring the highest binding energy gain (between -10.0 and -9.4 kcal/mol) are shown in **Figure 5**. The top-scoring molecules satisfy the following criteria: (i) the content of heteroatoms that improve water solubility and can potentially ensure specific binding to the protein, and (ii) the potential for parallel or T-shaped π-stacking with surrounding residues.

**Figure 6:**
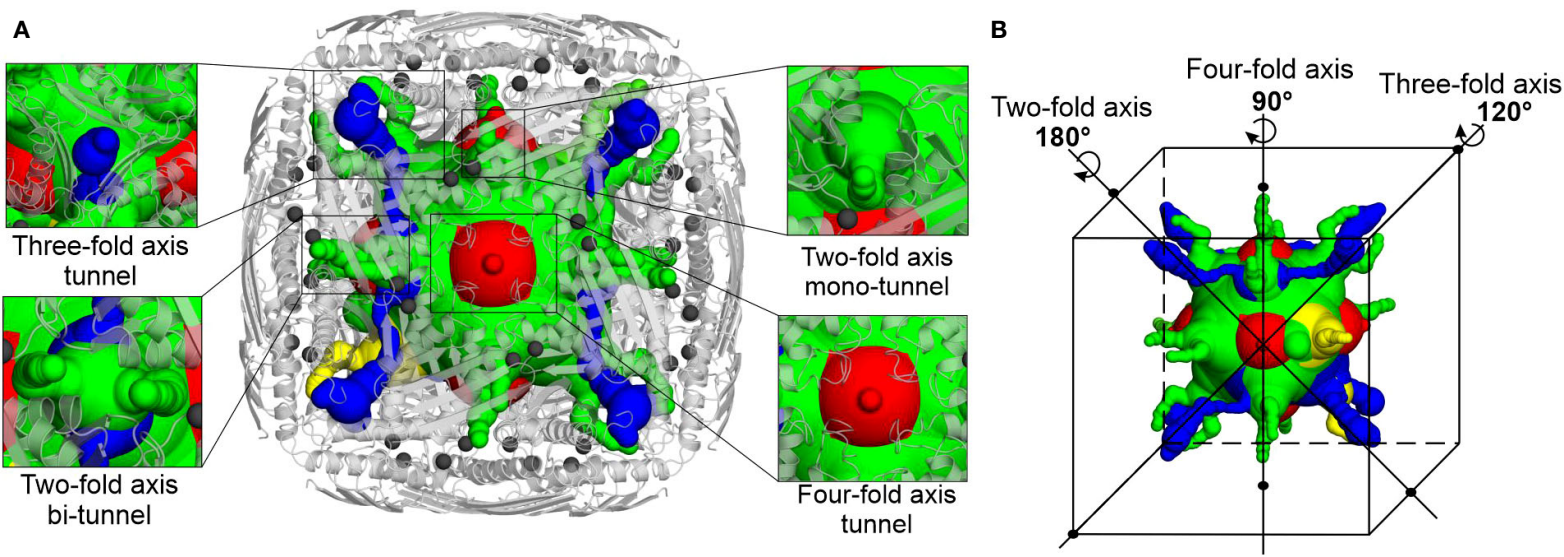
Molecular tunnels in the *Mt*HISN5 structure. **(A)** Tunnel distribution along symmetry axes; the tunnels along two-fold axes are subdivided into mono- and bi-tunnels. Two-fold axis mono-tunnels are depicted in yellow and bi-tunnels are depicted in green. Three-fold axis tunnels are blue and four-axis tunnels are red. Protein oligomer is shown as light gray cartoons and manganese ions are depicted as dim gray spheres. Note the proximity of the tunnel to the manganese-containing active sites. **(B)** Positioning of the tunnels along the symmetry axes; colors are the same as in **(A)**.

Intrigued by the highly symmetric and porous structure of *Mt*HISN5, we analyzed the molecular tunnels (in addition to the one presented in **Figure 4A**) that could let small molecules penetrate inside the enzyme. To this end, we studied the cryoEM *Mt*HISN5-unliganded structure in the *Caver Analyst 2.0* and *Caver 3.0.3 PyMol plugin* (Chovancova et al., 2012; Jurcik et al., 2018). Most of the tunnels are distributed along the 2-, 3-, and 4-fold axes (**Figure 6**). The average diameter of the tunnels along the 2-fold axes is large enough to allow infiltration only by very small molecules (< 60 Da), such as EDO, FMT, and water. Larger molecules, however, could permeate the inner cavity of *Mt*HISN5 through the tunnels along the 3-fold and 4-fold axes, which are approximately 2.5 times wider in diameter. Consistently, we identified TRS (121 Da) and CIT (192 Da, **Supplementary Figure S1D**) in the 3- and 4-fold tunnels, respectively.

### Genetic background of *M*tHISN5 and phylogenetic relationships with its homologs

*Mt*HISN5 (Uniprot ID: I3SDM5) is encoded by the *HISN5* gene (Ensembl: MTR_1g103820, Gene database: LOC25485215) located on chromosome 1. According to the Gene Database (Benson et al., 2013) and the *TargetP 2.0* webserver (Emanuelsson et al., 2000), *MtHISN5* contains an exon corresponding to a 76 amino acid residues long chloroplast transit peptide. The following intron separates the sequence of the genuine *Mt*HISN5 enzyme, which complies with previous observations for a canonical isoform in *A. thaliana*, HISN5B (Gene database: AT4G14910). This pattern suggests the fusion of the exon encoding the transit peptide with the exon encoding the enzyme sequence during evolution, which has been observed for several nuclear genes encoding chloroplast proteins (Wolter et al., 1988; Gantt et al., 1991; Stepansky and Leustek, 2006). A likely reason for the compartmentalization of the HBP is the interconnection with *de novo* purine metabolism that occurs in chloroplasts (and mitochondria). The HBP shares a precursor, 5-phosphoribosyl-1-pyrophosphate (PRPP) and an intermediate, aminoimidazolecarboximide ribonucleotide (AICAR), with *de novo* purine metabolism (Smith and Atkins, 2002; Witte and Herde, 2020). The obtained *Mt*HISN5 structures also allowed us to investigate the two transcript isoforms, X1 which is 1300 nt long, and X2 (1227 nt) in *M. truncatula*. The isoform X2 lacks the 5^th^ exon, which would result in a protein missing the loop-β7-loop fragment (residues Asp185 to Gln209). Therefore, it is very unlikely that the isoform X2 is expressed as a functional enzyme.

**Figure 7:**
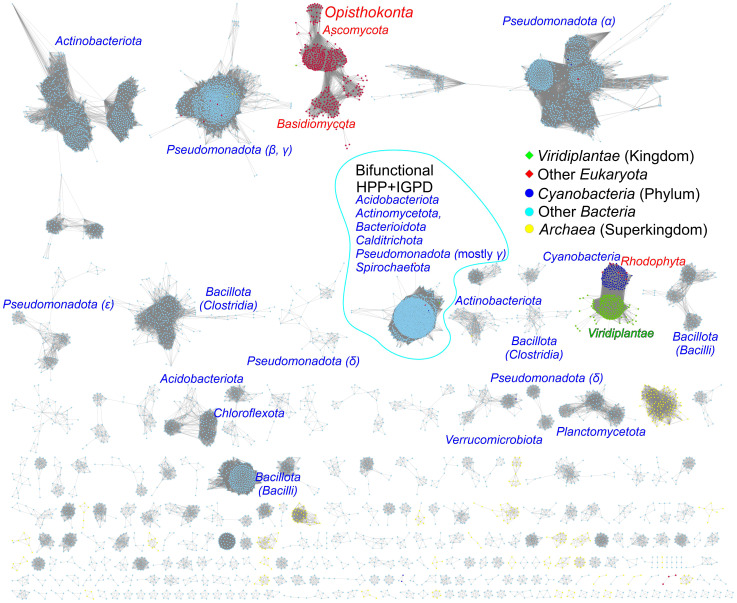
Sequence similarity network for the InterPro Family IPR000807. Plant and green algae HISN5 enzymes derive directly from *Cyanobacteria*. Red algae (*Rhodophyta*) constitute one group with *Cyanobacteria*. Fungal sequences (*Ascomycota* and *Basidiomycota*) are separated from other eukaryotic sequences. Bifunctional IGPD + HPP sequences from bacteria form a large group. Most bacterial and archaeal sequences are scattered in the diagram. Dots represent prokaryotes, diamonds represent eukaryotes, and the edges between them represent similarities.

To assess the similarity between prokaryotic and eukaryotic IGPD/HISN5 enzymes, we analyzed 12 710 sequences from the InterPro family IPR000807 by calculating a sequence similarity network (SSN, **Figure 7**). The result showed a close relationship between plants (*Viridiplantae*), green algae (*Chlorophyta*), and *Cyanobacteria* suggesting that plant HISN5s derive from cyanobacterial IGPDs. This is consistent with the endosymbiotic theory. To verify this observation, we generated a phylogenetic tree based on homologous sequences of *Mt*HISN5 (**Supplementary Figure S2**). The tree also showed a close relationship between the plant and cyanobacterial IGPDs, supporting the cyanobacterial origin of plant HISN5 enzymes. In contrast, we have recently shown that plant *HISN2* and *HISN6* are distant homologs of their cyanobacterial counterparts and are likely to have been acquired by HGT (Witek et al., 2021; Rutkiewicz et al., 2023).

The analysis also revealed a relatively large group of bacterial bifunctional enzymes presenting IGPD and HPP activity (**Figure 7**). Previous analyses of the phylogenetic origin of bacterial bifunctional *hisB*, i.e., *hisNB*, genes were limited to the classes of *γ-* and *ϵ-Proteobacteria* (Brilli and Fani, 2004; Kinateder et al., 2023). Our results suggest that the presence of fused genes occurs also in other bacterial phyla, providing new models for *his* genes’ evolution, especially interesting when combined with novel approaches for enzyme functional annotation (Kinateder et al., 2024). These bifunctional enzymes exist in *Acidobacteriota* (*Acidobacteria*)*, Actinomycetota* (*Actinobacteria*)*, Bacteroidota* (*Bacterioidetes*)*, Calditrichota* (*Calditrichaeota*)*, Pseudomonadota* (*γ-Proteobacteria*) and *Spirochaetota* (*Spirochaetes*); named according to the recent nomenclature update by the International Code of Nomenclature of Prokaryotes (Oren and Garrity, 2021) (former names are in parentheses).

In general, the majority of bacterial sequences present high sequence variability between phyla, which is much more significant than, for example, within plants. Moreover, fungal IGPD sequences are disconnected from other groups, suggesting that they differentiated early and have evolved in parallel. The conservation of HISN5 sequences in plant species, clearly distant from other kingdoms, suggests the possibility of reaching general-purpose herbicidal activity by potent HISN5 inhibitors. Such a potential is discussed in detail in the next chapter.

### Distinct features of plant HISN5 enzymes near the active site provide guidelines for inhibitor design

**Figure 8:**
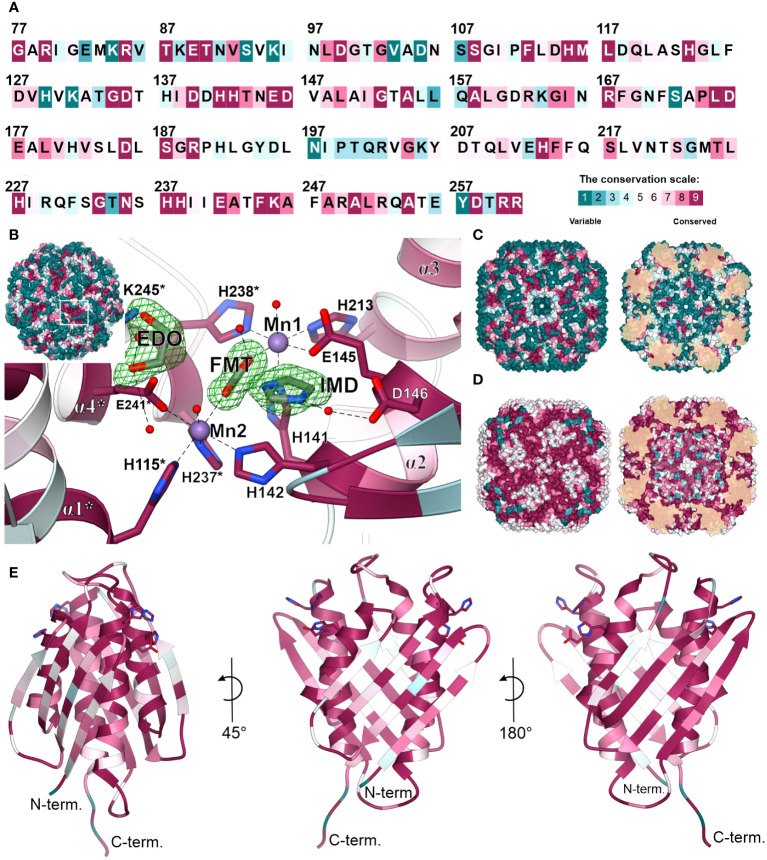
Residue conservation mapped on *Mt*HISN5. **(A)** Color-coded residue conservation of *Mt*HISN5 residues compared to all kingdoms based on the multiple sequence alignment (MSA); the color key given in the bottom-right corresponds to all panels in this Figure. **(B)** A close-up view of the conserved active site. Residues with the highest conservation score (all kingdoms) are involved in Mn^2+^ coordination and in the binding of small molecules in our structures. Red balls represent water molecules; formic acid (FMT), imidazole (IMD), and ethylene glycol (EDO) are in stick representation and contoured with polder electron density maps at the 10.0, 7.7, and 7.0 σ levels, respectively. **(C)** The outer surface (left) and inner surface (right) residue conservation based on the MSA calculated for all kingdoms. **(D)** Residue conservation among plants. Notice patterns at the interfaces on the outer surface of the protein. **(E)** The *Mt*HISN5 subunit is colored according to residue conservation in plants. The exposed side chains are residues that coordinate manganese ions.

The overall fold of IGPD enzymes, including their 24-meric assembly, is highly conserved between kingdoms of life, even though sequence conservation varies strongly. More precisely, *Mt*HISN5 sequence shares 88% identity with *A. thaliana* HISN5B (*At*HISN5B; 89% with *At*HISN5A), 45% with *Acanthamoeba castellani* (*Ac*IGPD), 40% with *Saccharomyces cerevisiae* (*Sc*IGPD), and 40% with *Staphylococcus aureus* (*Sa*IGPD). Therefore, we decided to perform a detailed analysis to pinpoint differences that could be exploited to ensure the selectivity of future inhibitors of plant HISN5 versus bacterial IGPD homologs. To obtain a perspective on the conserved and variable regions, we analyzed residue conservation using the *ConSurf* web server (Ashkenazy et al., 2016). Results of the Multiple Sequence Alignment (MSA) amongst all kingdoms reveal that the highest conservation score was assigned to residues forming HISN5 active sites and coordinating manganese ions: (i) Mn1 by His141, Glu145, His213, His238*, and (ii) Mn2 by His115*, His237*, Glu241*, His142 (**Figure 8A**). There were other residues located close to the active site area that were assigned the highest conservation rank and are also conserved in the aforementioned species, e.g., Asp146 or Lys245 that take part in ligand binding (**Figure 8B**) or Arg167 and Arg189 that stabilize the substrate’s phosphate group by weak hydrogen bonding (**Figure 3D**). Interestingly, residues comprising both β-sheets that are located on the outer and inner surfaces of the oligomer are rather variable across sequences from all kingdoms (**Figure 8C**). It is possible that this variability stems from the evolutionary pressure caused by operating in various environments, such as the bacterial and fungal cytosol and chloroplast stroma of plants.

When only protein sequences within the plant kingdom are considered, residues forming β-sheets on the outer and inner HISN5 surfaces are more conserved (**Figure 8D**). Still, more variability is observed at the outer surface compared to the inner (**Figure 8E**), suggesting some evolutionary pressure to maintain the environment in the hollow core of HISN5. In addition to the active site residues, the highest conservation was observed at the inter-subunit interfaces. Residues forming the central α-helical bundle are also highly conserved in plants (**Figure 8E**). Interestingly, several loops are also conserved, which is common not only for loops involved in substrate recognition, but also for those shaping internal tunnels, channels, or voids (Kress et al., 2018).

**Figure 9:**
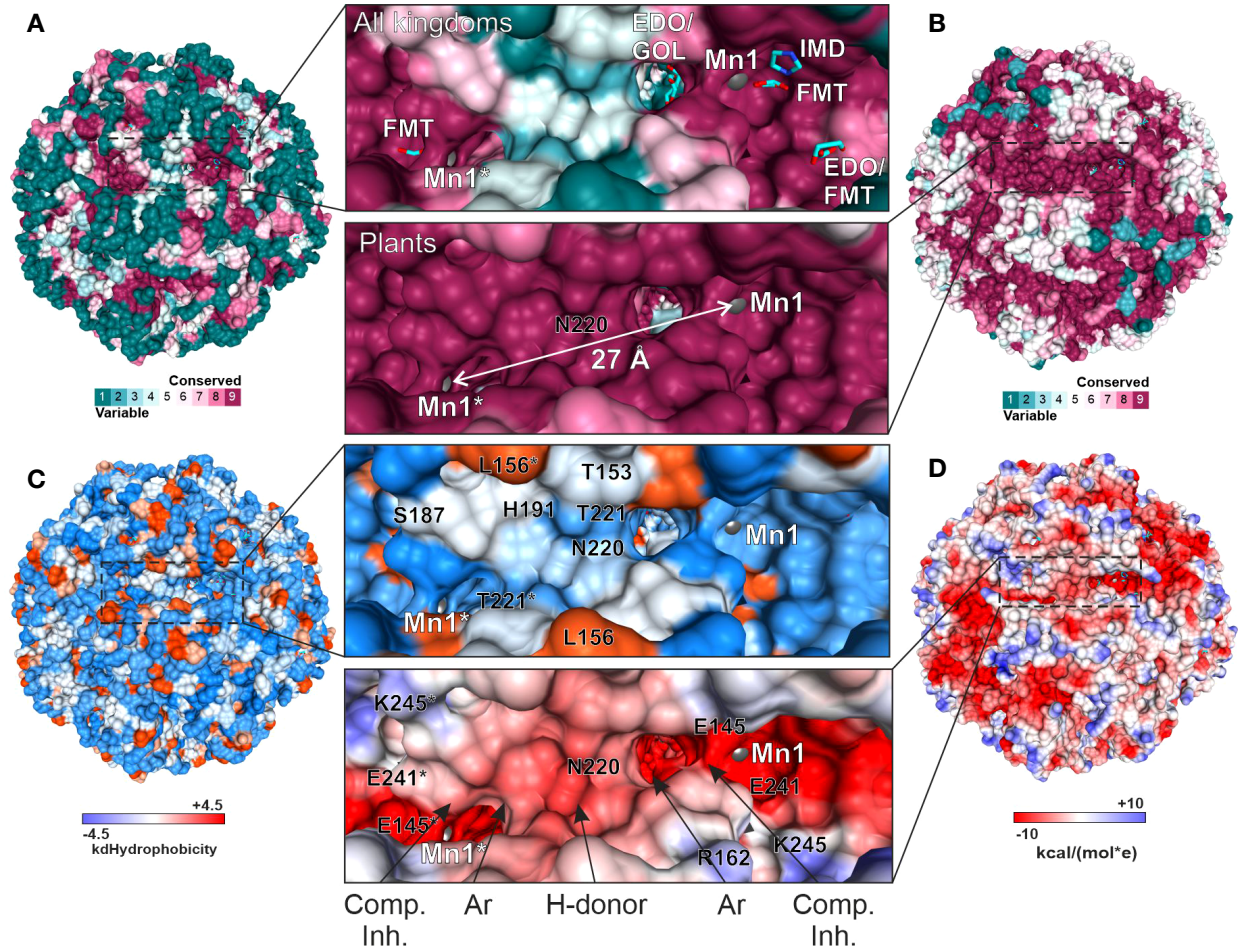
Depiction of the cleft that connects two active sites and is highly conserved in plants. All the panels show the same perspective. Panels **(A)** and **(B)** show the residue conservation scores for all organisms and plants, respectively. **(A)** contains the ligands bound in the crystal structures. The same residues in **(B)** are dark pink, meaning they are highly conserved in plants. The distance between Mn1 and Mn1* is indicated in **(B)**. **(C)** depicts hydrophobicity in the region of interest. **(D)** presents coulombic potential that indicates a majority of negatively charged residues. The proposed scaffold of symmetric inhibitors reaching two active sites includes competitive inhibitor (Comp. Inh.), aromatic (Ar), and hydrogen bond donating (H-donor) moieties.

We then compared the *ConSurf* data obtained for all kingdoms and exclusively for plants to identify a surface region in the vicinity of the active site that would be conserved in plants but vary in other kingdoms (**Figures 9A, B**). The goal was to propose a development pathway that would ensure both selectivity for plant HISN5 sequences and high potency, which will be crucial for designing HISN5 inhibitors and the subsequent development of herbicides. In this context, the cleft near Thr153, Ser187, His191, Asn220, and Thr221 (**Figure 9C**) is the most interesting, being variable in other species (**Figure 9A**) and highly conserved in plants (**Figure 9B**). This cleft connects active sites of two *Mt*HISN5 subunits (the Mn1-Mn1* distance is ~27 Å). The cleft is long and intrinsically symmetric, as it is crossed by one of the 2-fold axes. It is also rather hydrophilic and negatively charged (**Figures 9C, D**, respectively).

A common compound optimization method involves linking molecules that bind to a target at separate sites (Kirsch et al., 2019). The most versatile linkers are oligoethylene glycol chains. However, neither ethylene glycol molecules nor polyethylene glycol (PEG) fragments were identified in our structures, despite their use at high concentrations. This is consistent with the negative charge in the cleft center, which makes it predisposed to bind H-bond donors. However, PEG is an H-bond acceptor, except for the terminal hydroxyl groups. Therefore, we conducted another VS campaign focused on the cleft to find more suitable linkers between the two active sites. This time, however, we narrowed the screening library to include only more polar compounds (logP ≤ 2, ~1.3 mln molecules). The estimated binding energies were weaker than those obtained by VS in the active site (-8.1 vs. -10 kcal/mol, **Supplementary Figure S3**). Nonetheless, interesting common features became apparent in the top-scoring molecules. First, the fragment near the 2-fold axis between Pro190 and Pro190* appears prone to bind hydrophilic, preferably aliphatic, and six-membered-ring moieties, whereby secondary amines are H-bond donors. In contrast, more hydrophobic moieties tend to bind between the apolar parts of the Arg189 and Thr221* side chains, which are closer to the active sites. It is very important to note that targeting this cleft will enable the design of symmetric inhibitors that reach two active sites simultaneously. Such a multivalency can enhance the overall binding affinity and selectivity, reducing the likelihood of interactions with off-target proteins and minimizing side effects. Based on our experimental, comparative, and computational approaches, the scaffold which would best correspond to the HISN5 pharmacophore, would include competitive inhibitor (Comp. Inh.) moieties, at the poles of the molecule (**Figure 9D**). Next, aromatic moieties would connect to the molecule center that would be created by a symmetric and polar moiety with H-bond donors. Importantly, the competitive inhibitor moiety does not need to be a substrate/product or a transition state analog but could also contain carboxylate and imidazole moieties, as based on our crystal structures.

### *Mt*HISN5 activity measurements using isothermal titration calorimetry

**Figure 10:**
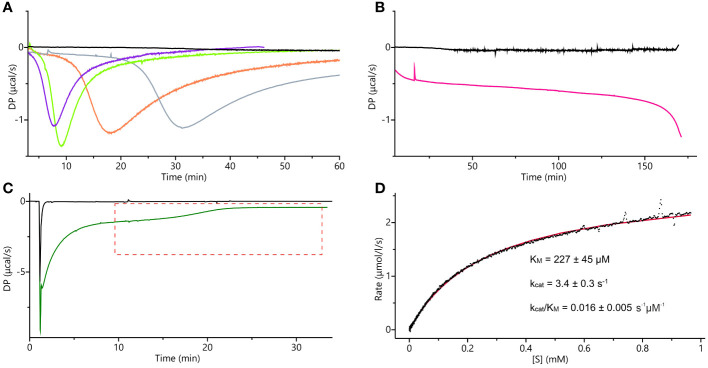
ITC measurements. **(A)** ITC-SIM superimposed raw data plots obtained after the blank experiment; injection of 30 µL of 0.5 mM commercial IGP (cIGP) into the cell containing buffer (black), 10-µL injection of 0.5 mM cIGP (final concentration 24 µM) into the cell with 10 nM *Mt*HISN5 (violet), 30-µL injection of 0.5 mM cIGP (final 65 µM) into the cell with 10 nM *Mt*HISN5 (green), 35 µL injection of 1.7 mM cIGP (final 253 µM) into the cell with 5 nM *Mt*HISN5 (orange), injection of the *Mt*HISN5 to the final concentration of 12 nM into the cell with 800 µM of cIGP (gray). **(B)** Injection of *Mt*HISN5 at a final concentration of 15 nM into the cell with 2 mM cIGP (magenta) and the blank (buffer to the substrate) experiment (black). **(C)** Representative raw ITC-SIM data (green line) after injection of 3.8 µL of 48 µM enzyme (to the final concentration in the cell of 901 nM) into eIGP at 3.4 mM concentration. DP values in the area marked with the red, dashed lines were converted into the rates and plotted against the substrate concentration; the black curve shows raw data from the blank experiment (buffer to reaction mixture). **(D)** The rates plotted against the substrate concentration were fitted with the Michaelis-Menten equation (red line), and the given parameters were calculated as an average of the values obtained from three separate experiments.

The supply of IGP is limited, and only 1-mg packages are currently available. Previously used absorption-based methods require large quantities of IGP as one needs to prepare 8 to 12 separate solutions, each containing a different substrate concentration, to obtain a single set of experimental data (Hawkes et al., 1995). Therefore, we decided to adopt and adjust the isothermal titration calorimetry single-injection method (ITC-SIM (Wang et al., 2020)). ITC-SIM consumes only ~200 µL of substrate at saturating concentration (instead of 8-12 mL) to obtain the enzyme kinetics graph showing rate of reaction as a function of substrate concentration. Our first ITC trials were performed on the same cIGP that was used for the cryoEM experiments. Although other IGPD enzymes were assayed using cIGP from the same source, we were not able to determine kinetic parameters. We could only confirm that the enzyme was active, as the curves clearly showed an exothermic event when compared to blank experiments (**Figures 10A, B**). Nonetheless, these measurements provided interesting insights into the *Mt*HISN5 behavior with cIGP. The exothermic reaction occurred after a lag phase, whose length was directly proportional to the cIGP concentration. During the lag phase, the heat production was very low, whereas theoretically, one should observe saturation with the substrate at the beginning of the experiment (**Figure 10A**). When using 24, 65, 253, and 800 µM of cIGP, this lag lasted for about 5, 7, 12, and 25 minutes, respectively. Subsequently, the highest heat production was observed, reflecting the achievement of V_max_ under the given conditions. Interestingly, with 2 mM cIGP, the initial lag reached the maximum time that the PEAQ-ITC apparatus offers (170 min). This peculiar behavior can likely be attributed to the contamination of cIGP with IG2, as revealed by our cryoEM experiments. The presence of IG2 (which cannot be enzymatically converted to IAP) in cIGP was also observed by others (Saika et al., 1993; Bisson et al., 2015). Hence, it was impossible to saturate *Mt*HISN5 with the cIGP substrate because an increase of the *2R,3S* diastereoisomer concentration always elevated the amount of *2S,3S* (IG2), which is a competitive binder.

To eliminate IG2 in the substrate sample, we synthesized IGP enzymatically (hereafter referred to as eIGP). We utilized *Mt*HISN1-4 and *E. coli* inorganic pyrophosphatase, each purified separately by Ni^2+^ affinity chromatography. Each reaction was monitored by absorbance (**Supplementary Figure S4**). After the reaction with *Mt*HISN4 was completed, the mixture was run through the Ni^2+^ resin to eliminate the proteins. This approach yields stereochemically pure IGP owing to the stereoselectivity of the HBP enzymes. The obtained yield of eIGP synthesis was 80%.

The ITC-SIM data obtained with eIGP were significantly more informative than those obtained with cIGP (**Figure 10C**). Despite the strong buffer mismatch, deriving from the high salt content in the vacuum-concentrated eIGP sample (in SpeedVac) and a likely heat effect of protein-substrate initial interaction, it was possible to observe the V_max_ plateau and the substrate depletion curve. Those data allowed us to fit the Michaelis-Menten equation and calculate the kinetic parameters: K_M_ = 227 ± 45 µM, *k*_cat_ = 3.4 ± 0.3 s^-1^ (**Figure 10D**). The K_M_ value reported for *At*HISN5 was 170 µM (Tada et al., 1995; Bisson et al., 2015), while HISN5 enzymes in crops exhibit a wide K_M_ range of 49 and 83 µM in *Triticum aestivum* (germ wheat and mature wheat, respectively), 600 µM in barley (*Hordeum vulgare*), up to 1.7 mM in oat, *Avenia sativa* (Wiater et al., 1971c). As for the *k*_cat_ value, according to our best knowledge and the BRENDA Enzymes database (Chang et al., 2021), only *k*_cat_ = 1400 for *M. tuberculosis* IGPD has been reported (Ahangar et al., 2013). Importantly, when comparing kinetic parameters from different studies, one should bear in mind different measurement conditions. Concentration of eIGP in the reaction buffer (optimal for enzymes synthesizing the substrate) results in relatively high salt content in the *Mt*HISN5 reaction (200 mM KCl and 100 mM NaCl), which may lower *k*_cat_. Nonetheless, the presented ITC-SIM enabled us to measure the kinetics for *Mt*HISN5 and obtain K_M_ and *k*_cat_ values consuming ~200 µL of saturating (3 mM) eIGP per one replicate. Such amounts are significantly lower than those required by former methods (Hawkes et al., 1995).

## Conclusions and outlook

For the past two decades, there has been an increase of interest in deciphering plant HBP, first from genetic, and then structural aspects. Previous methodological limitations in genetics, molecular, and structural biology have been overcome. The advent of new molecular tools, e.g., CRISPR-Cas9 (clustered regularly interspaced palindromic repeats)/Cas9-mediated genome editing), has proved that the lack of auxotrophic mutants is no longer a problem. Recently, a model liverworth, *Marchantia polymorpha*, was established as a eukaryotic *his* auxotrophic system for studying biocontainment and transformant selection without the need for antibiotics (Fukushima and Kodama, 2022). For many years, crystallography has been the only way to study the structures of plant HBP enzymes in detail (Ruszkowski and Dauter, 2016, Ruszkowski and Dauter, 2017; Ruszkowski, 2018; Witek et al., 2021; Rutkiewicz et al., 2023). The same was true for HISN5 (Glynn et al., 2005a; Bisson et al., 2015, Bisson et al., 2016) but now cryoEM will help to solve the experimental structures of plant HISN5 complexes with small molecules, including herbicide candidates.

This work also provides insights into *Mt*HISN5 phylogenetic relations with its prokaryotic and eukaryotic homologs, indicating that plant HISN5 sequences derive from *Cyanobacteria*, which is consistent with the endosymbiotic theory. Computational tools helped us point out highly conserved residues in plant HISN5s and map them onto the protein structure obtained experimentally. The highest conservation scores referred to the residues contributing to substrate binding and to those located at the intersubunit interfaces. Using both experimental and computational tools, we identified hot-spots that can interact with small molecules. Crystal and cryoEM structures allowed the identification of numerous ligands bound to *Mt*HISN5, i.e., FMT, EDO, IMD, TRS, CIT, GOL, ACT, PEG, Na^+^, Cl^-^, and SO_4_^2-^ (waters and Mn^2+^ excluded). None of the ligands significantly affected the global conformation of the enzyme. VS indicated molecules that could potentially bind to the *Mt*HISN5 surface. *In silico* analyses allowed us to characterize tunnels of different lengths and diameters, through which small molecules can permeate to the HISN5 central void and bind to the inner surface. Although with current data it is impossible to determine whether these tunnels are relevant for catalysis, novel plant HISN5 inhibitors could partially bind there, resulting in increased potency and selectivity.

HISN5 has been a target for herbicide design for decades, but no HISN5-specific herbicide is currently available (Klopotowski and Wiater, 1965; Wiater et al., 1971a, Wiater et al., 1971b). In the past years, there has been a noticeable growth of interest in targeting HISN5 (Bisson et al., 2015, Bisson et al., 2016; Rawson et al., 2018; Wang et al., 2021a, Wang et al., 2021b). The most common candidates for HISN5 inhibitors are triazole compounds such as amitrole or 2-hydroxy-3-(1,2,4-triazol-1-yl) propylphosphonate (C348) (Bisson et al., 2016). However, significant impediments have been affecting the evaluation of HISN5 activity owing to the scarcity of IGP on the market and the presence of competitively binding IGP diastereoisomer, IG2. With that in mind, we have adopted and adjusted ITC-SIM to measure HISN5 activity using enzymatically synthesized IGP. Therefore, this study sheds not only new light on plant HISN5, provides novel fragments of potential HISN5 inhibitors but also presents new methodologies for the rational design of HISN5 inhibitors with herbicidal activity.

## Materials and methods

### Sequence similarity network

Sequence similarity networks were calculated using the EFI-EST web server (Zallot et al., 2019). Input data consisted of 36 356 sequences belonging to the InterPro Family IPR000807 and were later reduced to 12 710 UniRef90 sequences. The calculations were based on sequences of 180 – 390 residues long and an alignment score of 80. Output results were visualized in *CytoScape 3.3* (Shannon et al., 2003).

### Cloning, expression, and purification

The coding sequence (CDS) of the *Mt*HISN5 gene was retrieved from the NCBI database (entry XP_013469848.1). The CDS was PCR-amplified using the primers *Mt*HISN5-Nt77-F (TACTTCCAATCCAATGCCGGTGCTAGAATTGGAGAGATGAAAAGG) and *Mt*HISN5-CtFL-R (TTATCCACTTCCAATGTTAACTACGCGACAGAACCCCTTTTGAA). The PCR product was purified and cloned into the expression plasmid pMCSG68 using the ligase-independent cloning (LIC) method (Kim et al., 2011). The final *Mt*HISN5 construct in this work was N-terminally truncated at Ser70 to obtain a higher yield of expression and enzyme stability compared to other tested constructs, truncated at residues 15, 30, 45, and 56. Because of the problematic purification of the enzyme fused with the His-tag, we deleted the tag entirely and instead inserted the 70-76 region of the original *Mt*HISN5 sequence using the polymerase incomplete primer extension (PIPE) method (Klock et al., 2008). The primers used for the PIPE method were *Mt*HISN5-Nt70-delHT-F (GAAGGAGATATACATATGCAACTTTCCCATATTGACTCAGGTGC) and *Mt*HISN5-Nt70-delHT-R (CATATTGTTATATCTCCTTCTTAAAGTTAAACAAATATTATTTTCTAGAGGGG). The cloning correctness was confirmed by DNA sequencing. Overexpression was carried out in BL21 Gold *E. coli* cells (Agilent Technologies) in an LB medium containing 150 μg/mL ampicillin. The cultures were grown at 37°C and shaken at 180 rpm. When OD_600_ reached 1.0, the temperature was lowered to 18°C, and the overexpression was induced using 0.5 mM isopropyl-D-thiogalactopyranoside (IPTG) and went on for 18 h; MnCl_2_ at 10 mM final concentration was added after IPTG. The cultures were centrifuged at 5000 × g for 15 min at 4°C and the sediment was suspended in 30-35 mL of purification buffer (40mM Tris–HCl pH 8.0, 40 mM NaCl, 4 mM Mn^2+^, 0.4 mM EDTA) and frozen at -75°C for purification.

The cells were disrupted by sonication (5 min with intervals for cooling), and cell debris was removed by centrifugation at 25,000 × g for 30 min at 4°C. We then followed a method of purification with DEAE-cellulose and NaCl gradient described by Glynn (Glynn et al., 2005b), however, *Mt*HISN5 was not present in the increasing NaCl gradient but in the first flow-through. Therefore, we decided to skip the gradient procedure and modify the method. After centrifugation at 25,000 × g, *Mt*HISN5 was precipitated by the addition of 1.7 M ammonium sulfate. The precipitate was collected by centrifugation at 20,000 × g for 15 min at 4°C and dissolved in 2 mL of purification buffer. The protein solution was filtered through a 0.45 µm syringe filter and 2.0 mL were loaded onto a Superose 6 column previously equilibrated with the purification buffer for size-exclusion chromatography (SEC). After elution, fractions containing *Mt*HISN5 were combined, concentrated to ~ 2.0 mL using Amicon Ultra Centrifugal Filters (Merck), and loaded onto a Superdex 200 column, also equilibrated with the purification buffer. Eluted fractions containing pure *Mt*HISN5 (based on SDS-PAGE) were pooled and concentrated.

The concentration was assessed by two methods because of the low extinction coefficient (ϵ = 4470 M^-1^ cm^-1^) and possible contamination with small molecules absorbing at 280 nm, thus creating a false-positive result of a higher than actual protein concentration (>50 mg/mL). The initial measurements were conducted at λ = 280 nm. The second spectrophotometric measurement was performed using the Bradford method (Bradford, 1976). Crystal structures were obtained from concentrations of 11-15 mg/mL, whereas cryoEM structures were obtained from 1 mg/mL.

*Mt*HISN1 and *Mt*HISN2 for enzymatic synthesis of IGP were produced as described previously (Ruszkowski, 2018; Witek et al., 2021), omitting TEV cleavage and dialysis. *Mt*HISN3 (N-terminally truncated at residue 42), *Mt*HISN4 (truncated at residue 48), and *E. coli* inorganic pyrophosphatase were obtained following the procedure described for *Mt*HISN1, with SEC directly after elution from Ni-NTA resin. The following primers were used to amplify *Mt*HISN3 and *Mt*HISN4 CDSs: MtHISN3-Nt42-F TACTTCCAATCCAATGCCTCTCCACCTTCAATTTCAATGCTCCGTTCAATTC, MtHISN3-CtFL-R TTATCCACTTTCCAATGTTAAGCCACTGAGACCTTTTGCTGGTTATGC, MtHISN4-Nt48-F TACTTCCAATCCAATGCCACTTCTATATGATTCTGTTGTGACTTTGCTTGATTATGGTG, and MtHISN4-CtFL-R TTATCCACTTCCAATGTTAGATTCGGACTTCTATGCCTTCATTCAACAAATGTTCTTT.

### Crystallization, X-ray data collection, and processing

*Mt*HISN5 was crystallized using the vapor diffusion method (hanging drop) and all crystallizations were set up manually at 20°C. The structure at 1.55 Å was obtained from the Morpheus screen (Gorrec, 2009) in Molecular Dimensions (MD1-46), condition 2-27 (G3) containing 0.1 M carboxylic acids (0.2 M sodium formate, 0.2 M ammonium acetate, 0.2 M sodium citrate tribasic dihydrate, 0.2 M potassium sodium tartrate tetrahydrate, 0.2 M sodium oxamate), 0.1 M imidazole, 4-morpholine-ethane-sulfonic acid (MES) adjusted by ratio to pH 6.5 and 30% v/v precipitant mix (40% v/v glycerol and 20% v/v PEG 4000).

The ShotGun Screen (SG-1 MD1-89-ECO), from Molecular Dimensions (Fazio et al., 2014) supplemented with 15% glycerol yielded the structure at 1.69 Å resolution. The measured crystals were obtained by mixing 2.0 µL of the *Mt*HISN5 solution with 2.0 µL of the condition 2-11 (E11) containing 2.0 M sodium formate, 0.1 M sodium acetate pH 4.6.

The structure at 2.2 Å was obtained based on the Morpheus screen (Gorrec, 2009) (MD1-46) in condition 2-31 (G7) containing 0.1 M carboxylic acids (0.2 M sodium formate, 0.2 M ammonium acetate, 0.2 M sodium citrate tribasic dihydrate, 0.2 M potassium sodium tartrate tetrahydrate, 0.2 M sodium oxamate), sodium 4-(2-hydroxyethyl)-1-piperazineethanesulfonic acid (HEPES), 3-(*N*-morpholino) propane sulfonic acid (MOPS) at pH 7.5, and 30% v/v precipitant mix (40% v/v glycerol and 20% v/v PEG 4000). The crystals were cryoprotected with 15% glycerol, vitrified in liquid nitrogen, and stored for data measurement. Diffraction data for structures at 1.55 and 1.69 Å were measured at the P13 beamline at the PETRA III synchrotron in Hamburg, Germany. The diffraction data for the structure at 2.2 Å were measured using an in-house X-ray diffractometer, Rigaku XtaLAB Synergy-R. All datasets were processed using the *XDS Package* (Kabsch, 2010). Data statistics are summarized in **Table 1**.

**Table 1:** Diffraction data and refinement statistics.

	*Mt*HISN5 (1.55Å) PDB ID: 8QAW	*Mt*HISN5 (1.69 Å) PDB ID: 8QAX	*Mt*HISN5 (2.2 Å) PDB ID: 8QAY
Diffraction source	PETRA III, Beamline P13, DESY Hamburg	PETRA III, Beamline P13 DESY Hamburg	Rigaku XtaLAB Synergy-R IBCH PAS Poznan
Wavelength (Å)	0.9762	0.9763	1.5418
Temperature (K)	100	100	100
Rotation range per image (°)	0.1	0.1	0.2
Total rotation range (°)	360	240	135
Space group	*R*3	*I*4	*R*3
*a*, *b*, *c* (Å)	137.5, 137.5, 265.6	120.5, 120.5, 183.0	137.6, 137.6, 265.8
Mosaicity (°)	0.056	0.116	0.169
Resolution range (Å)/ highest resolution shell	58 – 1.55/1.65-1.55	80 – 1.69/1.79 – 1.69	80 – 2.20/2.26 – 2.20
No. of unique reflections	272024	145061	93797
Completeness (%)	99.9/99.5	99.9/99.2	98.8/95.0
Redundancy	10.29	9.13	2.99
I/σ(I)	11.9/1.8	21.6/1.1	5.7/1.2
*R*_meas_ (%)	11.0/102.1	5.6/215.1	23.4/134.7
*CC1/2* (%)	99.7/83.3	99.9/57.3	98.6/47.0
No. of reflections: working/test set	272 024/1089	145 061/1001	93797/932
*R_work_*/*R*_free_	0.129/0.161	0.174/0.198	0.189/0.231
No. of non-H atoms: Protein/Ligand/Water	11518/263/1334	8572/88/498	11536/176/777
R.m.s. deviations: Bonds (Å)/Angles (°)	0.005/0.783	0.006/0.808	0.007/0.931
Ramachandran plot: Most favored/allowed/outliers (%)	96.2/3.8/0.0	95.8/4.2/0.0	95.6/4.4/0.0
Average B-factor: Protein/water/ligands (Å^2^)	29.3/45.8/50.9	41.5/47.2/44.8	36.7/40.4/47.8

### Determination and refinement of the crystal structures

The crystal structure of *Mt*HISN5 was solved using molecular replacement based on the structure of *A. thaliana* HISN5 (PDB ID: 4MU0) in *PHASER* (Mccoy et al., 2007). The initial model was built using *Phenix.Autobuild* (Terwilliger et al., 2008). The *ACHESYM* server was used to rearrange the model within the unit cell (Kowiel et al., 2014). Automatic model refinement was performed in *Phenix.Refine* (Afonine et al., 2018) and manual corrections were conducted in *COOT* (Emsley et al., 2010). For generation of ligand restraints, *Phenix.eLBOW* was used (Moriarty et al., 2009). The structure at 1.55 Å was refined anisotropically, and structures at 1.69 Å and 2.2 Å were refined isotropically with translation-libration-screw (TLS) parameters. The refinement statistics are included in **Table 1**.

### CryoEM data collection

Preparation of the cryoEM samples and the data collection were performed at the SOLARIS CryoEM Facility (Kraków, Poland). Quantifoil TEM grids (300 mesh R1.2/1.3 copper) were glow-discharged on EM ACE200 (Leica Microsystems). The grids were then placed inside the FEI Vitrobot Mark IV chamber set to 100% humidity at 4°C and a total of 2.5 μl of the protein solution was applied (blotting parameters: blot time, 4 s; wait time, 10 s; drain time, 0 s; blot force, 0; blot total, 1). The grids were plunge-vitrified in liquid ethane and clipped in liquid nitrogen. The data were collected on a Titan Krios microscope (Thermo Fisher Scientific) operated at 300 kV, equipped with an FEI Falcon III (4k x 4k) direct electron detector. The detector operated in counting mode at 96 000× magnification, resulting in a calibrated physical pixel size of 0.86 Å px^−1^. The micrographs were acquired as 40-frame movies (the total dose of 40 e^−^ Å^−2^) at under-focus with a defocus range of −3.0 to −0.9 μm and 0.3 μm defocus step. A total of 954 and 3330 micrographs were collected for the *Mt*HISN5-unliganded and *Mt*HISN5-IG2 structures, respectively.

### CryoEM data processing

For the *Mt*HISN5-unliganded dataset, reconstruction of the map was performed in *Relion 3.1* within the *CCP-EM* package (Scheres, 2012; Wood et al., 2015). The contrast transfer function (CTF) was estimated in *CTFFIND4* (Rohou and Grigorieff, 2015); 871 micrographs passed the curation step. 2D references for particle picking were obtained after automatic picking of 8707 particles from 31 micrographs. Template-based picking identified 456759 particles which were extracted as 352-pixel boxes. Based on the 2D classification, 424472 particles (41 classes out of 50) were selected and subjected to 3D classification. Finally, 229366 good particles were used for high-resolution refinement with the *O* symmetry imposed. Per-particle CTF refinement and Bayesian particle polishing after the first refinement improved the resolution from 3.2 to 2.4 Å (gold-standard Fourier-shell correlation, GSFSC; without masking and postprocessing). The “shiny” particles were then imported into *Cryosparc 4.4* and used in non-uniform refinement which further improved the resolution to 2.25 Å.

The *Mt*HISN5-IG2 cryoEM data were processed in *Cryosparc 4.1* (Punjani et al., 2017). 2990 micrographs were selected based on the CTF fit resolution, ice thickness, and accumulated motion. Blob picking, followed by 2D classification was used to generate four templates for automatic picking. After inspection of the picks, 661163 particles were retained and extracted in 300-pixel boxes for 2D classification (100 classes). The good 25 classes contained 605614 particles which were used to build the initial map and for high-resolution refinement. The best resolution (2.2 Å, GSFSC) was obtained using the non-uniform refinement protocol.

The molecular model of *At*HISN5 was placed into the map in *UCSF Chimera 1.15* (Pettersen et al., 2004) and the sequence was fitted using *Phenix.Autobuild* (Terwilliger et al., 2008). Manual corrections to the models were done in *Coot* (Emsley et al., 2010), between iterative rounds of automatic real-space model refinements in *Phenix.Refine* (Afonine et al., 2012). The latter also validated the model geometry and model-to-map correlation; details are listed in **Table 2**.

**Table 2:** CryoEM data and real-space refinement statistics.

	HISN5-unliganded	HISN5-IG2
PDB ID	7OJ5	8QAV
EMDB	EMD-12938	EMD-18305
Magnification (×)	96 000	96 000
Voltage (kV)	300	300
Electron exposure (e–/Å^2^)	40	40
Defocus range (μm)	−3.0 to −0.9	−3.0 to −0.9
Pixel size (Å)	0.86	0.86
Initial particle images (no.)	456759	661163
Final particle images (no.)	229366	605614
Resolution (gold-standard, Å)	2.25	2.23
FSC threshold	0.143	0.143
Map resolution range (Å)	2.10-3.25	1.95-3.50
B-factor for map sharpening	−110	113
Composition:		
Atoms	35587	35883
Protein residues	4440	4416
Water	931	1131
R. m. s. deviations:		
Bond (Å) (# > 4σ)	0.008 (0)	0.008 (0)
Angles (°)(# > 4σ)	1.004 (48)	1.188 (0)
Ramachandran plot (%):		
Outliers	0.55	0.55
Allowed	8.20	7.14
Favored	91.26	92.31
Rotamer outliers (%)	2.58	3.25
Cβ outliers (%)	0.00	0.00
Mean ADP (B-factors)		
Protein	30.58	19.58
Ligand	33.28	37.63
Water	26.70	20.45

### Virtual screening

All docking experiments were performed in *AutoDock Vina* (Trott and Olson, 2010) with the exhaustiveness = 8, using Python scripts to automate and parallelize the work. The receptor files were prepared with the *UCSF Chimera DockPrep* tool (Pettersen et al., 2004). VS in the active site was run using the library of lead-like molecules (3,344,603 in-stock compounds; 300-350 Da, logP ≤ 3.5) downloaded from the ZINC15 database (Sterling and Irwin, 2015) in December 2021. The structure in the *I*4 space group (1.69 Å resolution) was used as the receptor. The search box was centered at -2, 44, 63 Å (x, y, z) and measured 31 × 23 × 27 Å. To propose potential binders in the cleft conserved in plants but variable in other kingdoms, we selected a subset from the lead-like library in the ZINC15 database (Sterling and Irwin, 2015) containing more soluble molecules of logP ≤2 (1,355,624 docking-ready files downloaded in March 2022). The unliganded cryoEM structure (PDB ID: 7OJ5) was used as the receptor. The search box with the dimensions of 20 × 20 × 23 Å was centered at 114, 115, 155 Å (x, y, z). All results were scored based on the calculated binding energy gain.

### Other software

Multiple sequence alignment and the analysis of residue variability were performed using the *ConSurf Server* (Ashkenazy et al., 2016). The multiple sequence alignment for both high (> 95%) and low (> 35%) sequence identity for homologs was built using the MAFFT algorithm. The homologs were collected from UNIREF90 by the CS-BLAST search algorithm. The calculations resulted in 54 unique sequences of high percent identity and 150 unique sequences of low identity. The conservation scores were assigned using the Bayesian method of calculation and the best-fit model of substitution for proteins.

The phylogenetic tree was constructed using BLAST (Altschul et al., 1997) pairwise alignments. Distances were calculated using Kimura’s method (Kimura, 1983), and the tree was built using the Neighbor-Joining method (Saitou and Nei, 1987). BLASTP search using *Mt*HISN5-Nt70 sequence as a query, resulted in 477 Swissprot records with sequence identity between 30 and 90%. Sequences with the lowest percent identity were chosen as an outgroup root (*Thermotoga* sp.). The tree was visualized in *IcyTree* (Vaughan, 2017).

Molecular tunnel analysis was conducted using *CAVER Analyst 2.0* (Jurcik et al., 2018) and *CAVER 3.0.3* (Chovancova et al., 2012) plugin for *PyMOL 2.4.0* software. To detect the molecular tunnels, we used the following starting point coordinates 129.834, 136.402, 130.468 (x, y, z), probe radius 1.4, shell radius 6.0, shell depth 4, clustering threshold 3.5, frame weighting coefficient = 1.0, frame clustering threshold = 1.0 and number of iterations = 12. Received tunnels and their distance from water molecules within active sites were depicted and calculated using *UCSF Chimera 1.15* (Pettersen et al., 2004) and *PyMOL* (Schrodinger).

All protein models and structural alignment using the Needleman – Wunsch algorithm and the BLOSUM-62 matrix were visualized using *UCSF Chimera 1.15* (Pettersen et al., 2004) and *UCSF ChimeraX 1.5* (Pettersen et al., 2021).

### Enzymatic synthesis of IGP

Enzymatic synthesis required *in vitro* reconstitution of five steps of the HBP leading to the formation of *2R,3S*-IGP. The reaction was conducted in a total volume of 2.75 mL at 295 K and absorbance was measured at λ = 290 nm. All enzymes used in the synthesis were His-tagged at the N-termini. All steps were conducted in the kinetic buffer (Tris-HCl 50 mM, pH 8.0; MgCl_2_ 4 mM; KCl 100 mM; NaCl 50 mM, TCEP 1 mM). The first step required 1 µM *Mt*HISN1, 40 µM *E. coli* pyrophosphatase, and 2 mM ATP. The mixture was blanked and the reaction was initiated using 2 mM PRPP. The reaction was conducted until a plateau was reached (approximately 50 min, absorbance reached 1.1, **Supplementary Figure S4A**). The mixture was blanked once again, and 1 µM *Mt*HISN2 was added. After 20 min when A = 2.0 (**Supplementary Figure S4B**), the mixture was blanked and 1 µM *Mt*HISN3, 5 µM *Mt*HISN4, and 5 mM L-glutamine were added simultaneously to start the last steps of enzymatic synthesis. After approximately 150 s, a decrease in absorbance was observed at 300 nm, indicating that PR-FAR was converted by *Mt*HISN4 into *2R,3S*-IGP (**Supplementary Figure S4C**). The mixture was incubated for 5 minutes on ice with 300 µL of Ni-NTA resin (GE Healthcare) and centrifuged at 2000 × g for 3 minutes. Notably, we tried purification using membrane filters to remove enzymes and potential intermediates, but we did not detect eIGP in the flowthrough, suggesting that eIGP was captured by the membrane. The resin was pre-equilibrated in binding buffer (Tris-HCl 50 mM, pH 8.0; NaCl 500 mM, imidazole 20 mM, TCEP 1 mM, 10% glycerol). The supernatant containing IGP was aspirated, transferred into a fresh Eppendorf tube, and kept on ice. The concentration of *2R,3S*-IGP was assessed using a glutamate assay kit (Sigma Aldrich). Glutamate was formed from glutamine by *Mt*HISN4, therefore its concentration of 1.6 mM stoichiometrically corresponded to a concentration of *2R,3S*-IGP, indicating 80% yield of eIGP synthesis.

### Isothermal titration calorimetry

The kinetics of *Mt*HISN5 were monitored using microcalorimetry (MicroCal PEAQ-ITC and MicroCal iTC200, Malvern). ITC-SIM has been used for this purpose (Wang et al., 2020). The reaction was conducted in SEC buffer (Tris-HCl 40 mM pH 7.8, NaCl 40 mM, MnCl_2_ 4 mM, EDTA 0.4 mM) at 30°C. The differential power (DP) of all ITC-SIM experiments was set to 10 µcal/mol and the stirring speed at 650 rpm. For experiments with cIGP (Santa Cruz Biotechnology, sc-218019), the protein in the cell was maintained at a low nanomolar concentration (5-10 nM), and the substrate in the syringe was added in one injection to the final concentration varying from 24 to 235 µM. Measurements were stopped when the baseline returned to the initial state, signaling substrate depletion. For one of the tested setups of the ITC-SIM experiment, a blank experiment was performed consisting of one injection of 30 µL of 0.5 mM cIGP into the cell containing the buffer, and the observation time was set to the maximum for the apparatus (10 000 s) to check the possibility of heat effect derived from the non-enzymatic substrate degradation. Additionally, an inverted ITC-SIM system was introduced to achieve a higher cIGP concentration (by circumventing the problem of the high heat of substrate dilution), where the substrate (in the cell) was kept at 0.8- and 2-mM concentrations and concentrated enzyme in the syringe (capped with 3 µL of the buffer to prevent the early leakage of the enzyme) was injected in 1 portion into the cell at a final concentration of 12 and 15 nM, respectively.

For the ITC-SIM experiments on eIGP, the substrate was concentrated using a SpeedVac. We maintained the concentration of eIGP in the cell in the range of 1.9-3.4 mM and injected the concentrated *Mt*HISN5 to its final concentration of 792-901 nM (a 3.8 µL aliquot of 42-48 µM enzyme, subunit concentration). Measurements were stopped ~10 min after the baseline returned to near the initial state, signaling total substrate depletion. The DP baseline was analyzed after the initial DP drop (coming from the buffer mismatch and initial protein-substrate interaction) and fitted to the ‘Enzyme Kinetics – Single Injection’ model within the MicroCal PEAQ-ITC analysis software. Briefly, the raw data were transformed into reaction rates and IGP concentrations and fitted to the Michaelis-Menten equation. The final kinetic parameters were calculated by averaging the values obtained from the three separate experiments.

## Data availability statement

The datasets presented in this study can be found in online repositories. The names of the repository/repositories and accession number(s) can be found below: Raw diffraction datasets are available from the Macromolecular Xtallography Raw Data Repository (MX-RDR; www.mxrdr.icm.edu.pl). The access is available through following DOIs: (i) MtHISN5-1.69A, https://doi.org/10.18150/XWLKP6; (ii) MtHISN5-1.55A, https://doi.org/10.18150/INUP4Q; (iii) MtHISN5-2.2A, https://doi.org/10.18150/FN6VJX. The crystal and cryo-EM structures are available in the Protein Data Bank (PDB, www.rcsb.org) and the Electron Microscopy Data Bank (EMDB; www.ebi.ac.uk/emdb). MtHISN5-unliganded: PDB 7OJ5, EMDB EMD-12938; MtHISN5-IG2: PDB 8QAV, EMDB EMD-18305; MtHISN5-1.55 Å: PDB ID 8QAW; MtHISN5-1.69 Å: PDB ID 8QAX; MtHISN5-2.20 Å: PDB ID 8QAY.

## Author contributions

WW: Data curation, Investigation, Methodology, Visualization, Writing – original draft. JS: Conceptualization, Investigation, Methodology, Writing – original draft. MRa: Methodology, Writing – original draft. MRu: Conceptualization, Funding acquisition, Methodology, Project administration, Supervision, Writing – review & editing.

## References

[B1] Adams E. (1954). The enzymatic synthesis of histidine from histidinol. J. Biol. Chem. 209, 829–846. doi: 10.1016/S0021-9258(18)65512-7 13192138

[B2] Afonine P. V. Grosse-Kunstleve R. W. Echols N. Headd J. J. Moriarty N. W. Mustyakimov M.. (2012). Towards automated crystallographic structure refinement with phenix.refine. Acta Crystallogr. D Biol. Crystallogr. 68, 352–367. doi: 10.1107/S0907444912001308 22505256 PMC3322595

[B3] Afonine P. V. Poon B. K. Read R. J. Sobolev O. V. Terwilliger T. C. Urzhumtsev A.. (2018). Real-space refinement in PHENIX for cryo-EM and crystallography. Acta Cryst. D 74, 531–544. doi: 10.1107/S2059798318006551 PMC6096492 29872004

[B4] Ahangar M. S. Vyas R. Nasir N. Biswal B. K. (2013). Structures of native, substrate-bound and inhibited forms of Mycobacterium tuberculosis imidazoleglycerol-phosphate dehydratase. Acta Crystallogr. D Biol. Crystallogr. 69, 2461–2467. doi: 10.1107/S0907444913022579 24311587

[B5] Akashi H. Gojobori T. (2002). Metabolic efficiency and amino acid composition in the proteomes of Escherichia coli and Bacillus subtilis. PNAS 99, 3695–3700. doi: 10.1073/pnas.062526999 11904428 PMC122586

[B6] Altschul S. F. Madden T. L. Schaffer A. A. Zhang J. H. Zhang Z. Miller W.. (1997). Gapped BLAST and PSI-BLAST: a new generation of protein database search programs. Nucleic Acids Res. 25, 3389–3402. doi: 10.1093/nar/25.17.3389 9254694 PMC146917

[B7] Ames B. N. (1957a). The biosynthesis of histidine - D-erythro-imidazoleglycerol phosphate dehydrase. J. Biol. Chem. 228, 131–143. doi: 10.1016/S0021-9258(18)70696-0 13475302

[B8] Ames B. N. (1957b). The biosynthesis of histidine - L-histidinol phosphate phosphatase. J. Biol. Chem. 226, 583–593. doi: 10.1016/S0021-9258(18)70840-5 13438843

[B9] Ames B. N. Mitchell H. K. (1955). The biosynthesis of histidine - imidazoleglycerol phosphate, imidazoleacetol phosphate, and histidinol phosphate. J. Biol. Chem. 212, 687–696. doi: 10.1016/S0021-9258(18)71007-7 14353870

[B10] Ashkenazy H. Abadi S. Martz E. Chay O. Mayrose I. Pupko T.. (2016). ConSurf 2016: an improved methodology to estimate and visualize evolutionary conservation in macromolecules. Nucleic Acids Res. 44, W344–W350. doi: 10.1093/nar/gkw408 27166375 PMC4987940

[B11] Beckie H. J. Busi R. Lopez-Ruiz F. J. Umina P. A. (2021). Herbicide resistance management strategies: how do they compare with those for insecticides, fungicides and antibiotics? Pest Manage. Sci. 77, 3049–3056. doi: 10.1002/ps.6395 33821561

[B12] Benson D. A. Cavanaugh M. Clark K. Karsch-Mizrachi I. Lipman D. J. Ostell J.. (2013). GenBank. Nucleic Acids Res. 41, D36–D42. doi: 10.1093/nar/gks1195 23193287 PMC3531190

[B13] Bisson C. Britton K. L. Sedelnikova S. E. Rodgers H. F. Eadsforth T. C. Viner R. C.. (2015). Crystal structures reveal that the reaction mechanism of imidazoleglycerol-phosphate dehydratase is controlled by switching mn(II) coordination. Structure 23, 1236–1245. doi: 10.1016/j.str.2015.05.012 26095028 PMC4509728

[B14] Bisson C. Britton K. L. Sedelnikova S. E. Rodgers H. F. Eadsforth T. C. Viner R. C.. (2016). Mirror-image packing provides a molecular basis for the nanomolar equipotency of enantiomers of an experimental herbicide. Angew Chem. Int. Edit 55, 13485–13489. doi: 10.1002/anie.201607185 PMC5113775 27717128

[B15] Bradford M. M. (1976). A rapid and sensitive method for the quantitation of microgram quantities of protein utilizing the principle of protein-dye binding. Anal. Biochem. 72, 248–254. doi: 10.1016/0003-2697(76)90527-3 942051

[B16] Brenner M. Ames B. N. (1971). “The histidine operon and its regulation,” in Metabolic Pathways, 3 ed. Ed. G. D. M. (Academic Press, New York).

[B17] Brilli M. Fani R. (2004). Molecular evolution of *hisB* genes. J. Mol. Evol. 58, 225–237. doi: 10.1007/s00239-003-2547-x 15042344

[B18] Burks D. Azad R. Wen J. Q. Dickstein R. (2018). The *medicago truncatula* genome: Genomic data availability. Funct. Genomics Medicago Truncatula: Methods Protoc. 1822, 39–59. doi: 10.1007/978-1-4939-8633-0_3 30043295

[B19] Cantin N. E. Negri A. P. Willis B. L. (2007). Photoinhibition from chronic herbicide exposure reduces reproductive output of reef-building corals. Mar. Ecol. Prog. Ser. 344, 81–93. doi: 10.3354/meps07059

[B20] Chang A. Jeske L. Ulbrich S. Hofmann J. Koblitz J. Schomburg I.. (2021). BRENDA, the ELIXIR core data resource in 2021: new developments and updates. Nucleic Acids Res. 49, D498–D508. doi: 10.1093/nar/gkaa1025 33211880 PMC7779020

[B21] Chovancova E. Pavelka A. Benes P. Strnad O. Brezovsky J. Kozlikova B.. (2012). CAVER 3.0: a tool for the analysis of transport pathways in dynamic protein structures. PLoS Comput. Biol. 8, e1002708. doi: 10.1371/journal.pcbi.1002708 23093919 PMC3475669

[B22] Del Duca S. Chioccioli S. Vassallo A. Castronovo L. M. Fani R. (2020). The role of gene elongation in the evolution of histidine biosynthetic genes. Microorganisms 8, ARTN 732. doi: 10.3390/microorganisms8050732 PMC7284861 32414216

[B23] Emanuelsson O. Nielsen H. Brunak S. Von Heijne G. (2000). Predicting subcellular localization of proteins based on their N-terminal amino acid sequence. J. Mol. Biol. 300, 1005–1016. doi: 10.1006/jmbi.2000.3903 10891285

[B24] Emsley P. Lohkamp B. Scott W. G. Cowtan K. (2010). Features and development of coot. Acta Crystallogr. D Biol. Crystallogr. 66, 486–501. doi: 10.1107/S0907444910007493 20383002 PMC2852313

[B25] Farina W. M. Balbuena M. S. Herbert L. T. Mengoni Gonalons C. Vazquez D. E. (2019). Effects of the herbicide glyphosate on honey bee sensory and cognitive abilities: Individual impairments with implications for the hive. Insects 10, 354. doi: 10.3390/insects10100354 31635293 PMC6835870

[B26] Fazio V. J. Peat T. S. Newman J. (2014). A drunken search in crystallization space. Acta Cryst. F 70, 1303–1311. doi: 10.1107/S2053230X1401841X PMC4188070 25286930

[B27] Frumkin I. Schirman D. Rotman A. Li F. Zahavi L. Mordret E.. (2017). Gene architectures that minimize cost of gene expression. Mol. Cell 65, 142–153. doi: 10.1016/j.molcel.2016.11.007 27989436 PMC5506554

[B28] Fujimori K. Ohta D. (1998). An Arabidopsis cDNA encoding a bifunctional glutamine amidotransferase/cyclase suppresses the histidine auxotrophy of a Saccharomyces cerevisiae his7 mutant. FEBS Lett. 428, 229–234. doi: 10.1016/S0014-5793(98)00535-3 9654139

[B29] Fukushima T. Kodama Y. (2022). Selection of a histidine auxotrophicMarchantia polymorpha strain with an auxotrophic selective marker. Plant Biotechnol. 39, 345–354. doi: 10.5511/plantbiotechnology.22.0810a PMC10240916 37283617

[B30] Furukawa A. Oikawa S. Harada K. Sugiyama H. Hiraku Y. Murata M.. (2010). Oxidatively generated DNA damage induced by 3-amino-5-mercapto-1,2,4-triazole, a metabolite of carcinogenic amitrole. Mutat. Res. 694, 7–12. doi: 10.1016/j.mrfmmm.2010.08.004 20732334

[B31] Gaines T. A. Busi R. Kupper A. (2021). Can new herbicide discovery allow weed management to outpace resistance evolution? Pest Manage. Sci. 77, 3036–3041. doi: 10.1002/ps.6457 33942963

[B32] Gantt J. S. Baldauf S. L. Calie P. J. Weeden N. F. Palmer J. D. (1991). Transfer of rpl22 to the nucleus greatly preceded its loss from the chloroplast and involved the gain of an intron. EMBO J. 10, 3073–3078. doi: 10.1002/embj.1991.10.issue-10 1915281 PMC453023

[B33] Glynn S. E. Baker P. J. Sedelnikova S. E. Davies C. L. Eadsforth T. C. Levy C. W.. (2005a). Structure and mechanism of imidazoleglycerol-phosphate dehydratase. Structure 13, 1809–1817. doi: 10.1016/j.str.2005.08.012 16338409

[B34] Glynn S. E. Baker P. J. Sedelnikova S. E. Levy C. W. Rodgers H. F. Blank J.. (2005b). Purification, crystallization and preliminary crystallographic analysis of Arabidopsis thaliana imidazoleglycerol-phosphate dehydratase. Acta Cryst. F 61, 776–778. doi: 10.1107/S1744309105022451 PMC1952351 16511155

[B35] Gorrec F. (2009). The MORPHEUS protein crystallization screen. J. Appl. Crystallogr. 42, 1035–1042. doi: 10.1107/S0021889809042022 22477774 PMC3246824

[B36] Gould F. Brown Z. S. Kuzma J. (2018). Wicked evolution: Can we address the sociobiological dilemma of pesticide resistance? Science 360, 728–732. doi: 10.1126/science.aar3780 29773742

[B37] Hall C. J. Mackie E. R. Gendall A. R. Perugini M. A. Soares Da Costa T. P. (2020). Review: Amino acid biosynthesis as a target for herbicide development. Pest Manag Sci. 76, 3896–3904. doi: 10.1002/ps.5943 32506606

[B38] Hawkes T. R. Thomas P. G. Edwards L. S. Rayner S. J. Wilkinson K. W. Rice D. W. (1995). Purification and characterization of the imidazoleglycerol-phosphate dehydratase of saccharomyces-cerevisiae from recombinant escherichia-coli. Biochem. J. 306, 385–397. doi: 10.1042/bj3060385 7887893 PMC1136533

[B39] Hilton J. L. Kearney P. C. Ames B. N. (1965). Mode of action of herbicide 3-amino-1,2,4-triazole(Amitrole) - inhibition of an enzyme of histidine biosynthesis. Arch. Biochem. Biophys. 112, 544–&. doi: 10.1016/0003-9861(65)90093-7 5326242

[B40] Hrbácková M. Dvorák P. Takác T. Tichá M. Luptovciak I. Samajová O.. (2020). Biotechnological perspectives of omics and genetic engineering methods in alfalfa. Front. Plant Sci. 11. doi: 10.3389/fpls.2020.00592 PMC7253590 32508859

[B41] Ingle R. A. (2011). Histidine biosynthesis. Arabidopsis Book 9, e0141. doi: 10.1199/tab.0141 22303266 PMC3266711

[B42] Jurcik A. Bednar D. Byska J. Marques S. M. Furmanova K. Daniel L.. (2018). CAVER Analyst 2.0: analysis and visualization of channels and tunnels in protein structures and molecular dynamics trajectories. Bioinformatics 34, 3586–3588. doi: 10.1093/bioinformatics/bty386 29741570 PMC6184705

[B43] Kabsch W. (2010). Xds. Acta Crystallographica Section D-Biological Crystallogr. 66, 125–132. doi: 10.1107/S0907444909047337 PMC2815665 20124692

[B44] Kepp K. P. (2020). Survival of the cheapest: how proteome cost minimization drives evolution. Q Rev. Biophys. 53, e7. doi: 10.1017/S0033583520000037 32624048

[B45] Kim Y. Babnigg G. Jedrzejczak R. Eschenfeldt W. H. Li H. Maltseva N.. (2011). High-throughput protein purification and quality assessment for crystallization. Methods 55, 12–28. doi: 10.1016/j.ymeth.2011.07.010 21907284 PMC3690762

[B46] Kimura M. (1983). The Neutral Theory of Molecular Evolution (Cambridge: Cambridge University Press). doi: 10.1017/CBO9780511623486

[B47] Kinateder T. Drexler L. Straub K. Merkl R. Sterner R. (2023). Experimental and computational analysis of the ancestry of an evolutionary young enzyme from histidine biosynthesis. Protein Sci. 32, e4536. doi: 10.1002/pro.4536 36502290 PMC9798254

[B48] Kinateder T. Mayer C. Nazet J. Sterner R. (2024). Improving enzyme functional annotation by integrating in *vitro* and in silico approaches: The example of histidinol phosphate phosphatases. Protein Sci. 33, e4899. doi: 10.1002/pro.4899 38284491 PMC10804674

[B49] Kirsch P. Hartman A. M. Hirsch A. K. H. Empting M. (2019). Concepts and core principles of fragment-based drug design. Molecules 24, 4309. doi: 10.3390/molecules24234309 31779114 PMC6930586

[B50] Kishore G. M. Shah D. M. (1988). Amino acid biosynthesis inhibitors as herbicides. Annu. Rev. Biochem. 57, 627–663. doi: 10.1146/annurev.bi.57.070188.003211 3052285

[B51] Klock H. E. Koesema E. J. Knuth M. W. Lesley S. A. (2008). Combining the polymerase incomplete primer extension method for cloning and mutagenesis with microscreening to accelerate structural genomics efforts. Proteins 71, 982–994. doi: 10.1002/prot.21786 18004753

[B52] Klopotowski T. Wiater A. (1965). Synergism of aminotriazole and phosphate on inhibition of yeast imidazole glycerol phosphate dehydratase. Arch. Biochem. Biophys. 112, 562–566. doi: 10.1016/0003-9861(65)90096-2 5880156

[B53] Kowiel M. Jaskolski M. Dauter Z. (2014). ACHESYM: an algorithm and server for standardized placement of macromolecular models in the unit cell. Acta Crystallographica Section D-Biological Crystallogr. 70, 3290–3298. doi: 10.1107/S1399004714024572 PMC4257622 25478846

[B54] Kress N. Halder J. M. Rapp L. R. Hauer B. (2018). Unlocked potential of dynamic elements in protein structures: channels and loops. Curr. Opin. Chem. Biol. 47, 109–116. doi: 10.1016/j.cbpa.2018.09.010 30292890

[B55] Kumar D. Jha B. Bhatia I. Ashraf A. Dwivedy A. Biswal B. K. (2022). Characterization of a triazole scaffold compound as an inhibitor of imidazoleglycerol-phosphate dehydratase. Proteins 90, 3–17. doi: 10.1002/prot.26181 34288118

[B56] Mano J. Hatano M. Koizumi S. Tada S. Hashimoto M. Scheidegger A. (1993). Purification and properties of a monofunctional imidazoleglycerol-phosphate dehydratase from wheat. Plant Physiol. 103, 733–739. doi: 10.1104/pp.103.3.733 12231975 PMC159043

[B57] Martin R. G. Berberich M. A. Ames B. N. Davis W. W. Goldberger R. F. Yourno J. D. (1971). “Enzymes and intermediates of histidine biosynthesis in Salmonella typhimurium,” in Methods in Enzymology: Metabolism of Amino Acids and Amines Part B. Eds. Tabor C. W. Tabor H. (Academic Press, New York), 3–44.

[B58] Mccoy A. J. Grosse-Kunstleve R. W. Adams P. D. Winn M. D. Storoni L. C. Read R. J. (2007). Phaser crystallographic software. J. Appl. Crystallogr. 40, 658–674. doi: 10.1107/S0021889807021206 19461840 PMC2483472

[B59] Miller L. L. Bale W. F. (1952). Factors affecting biosynthesis of plasma albumin and globulin fractions as studied with the aid of lysine-epsilon and histidine-2-C-14. Fed Proc. 11, 260–260.

[B60] Moriarty N. W. Grosse-Kunstleve R. W. Adams P. D. (2009). electronic Ligand Builder and Optimization Workbench (eLBOW): a tool for ligand coordinate and restraint generation. Acta Cryst. D 65, 1074–1080. doi: 10.1107/S0907444909029436 19770504 PMC2748967

[B61] Motta E. V. S. Raymann K. Moran N. A. (2018). Glyphosate perturbs the gut microbiota of honey bees. Proc. Natl. Acad. Sci. U.S.A. 115, 10305–10310. doi: 10.1073/pnas.1803880115 30249635 PMC6187125

[B62] Mueller-Harvey I. Bee G. Dohme-Meier F. Hoste H. Karonen M. Kölliker R.. (2019). Benefits of condensed tannins in forage legumes fed to ruminants: Importance of structure, concentration, and diet composition. Crop Sci. 59, 861–885. doi: 10.2135/cropsci2017.06.0369

[B63] Muralla R. Sweeney C. Stepansky A. Leustek T. Meinke D. (2007). Genetic dissection of histidine biosynthesis in Arabidopsis. Plant Physiol. 144, 890–903. doi: 10.1104/pp.107.096511 17434988 PMC1914156

[B64] Negri A. Vollhardt C. Humphrey C. Heyward A. Jones R. Eaglesham G.. (2005). Effects of the herbicide diuron on the early life history stages of coral. Mar. pollut. Bull. 51, 370–383. doi: 10.1016/j.marpolbul.2004.10.053 15757736

[B65] Oren A. Garrity G. M. (2021). Valid publication of the names of forty-two phyla of prokaryotes. Int. J. Syst. Evol. Microbiol. 71, 5056. doi: 10.1099/ijsem.0.005056 34694987

[B66] Petersen L. N. Marineo S. Mandala S. Davids F. Sewell B. T. Ingle R. A. (2010). The missing link in plant histidine biosynthesis: Arabidopsis myoinositol monophosphatase-like2 encodes a functional histidinol-phosphate phosphatase. Plant Physiol. 152, 1186–1196. doi: 10.1104/pp.109.150805 20023146 PMC2832243

[B67] Pettersen E. F. Goddard T. D. Huang C. C. Couch G. S. Greenblatt D. M. Meng E. C.. (2004). UCSF chimera - A visualization system for exploratory research and analysis. J. Comput. Chem. 25, 1605–1612. doi: 10.1002/jcc.20084 15264254

[B68] Pettersen E. F. Goddard T. D. Huang C. R. C. Meng E. E. C. Couch G. S. Croll T. I.. (2021). UCSF ChimeraX: Structure visualization for researchers, educators, and developers. Protein Sci. 30, 70–82. doi: 10.1002/pro.3943 32881101 PMC7737788

[B69] Punjani A. Rubinstein J. L. Fleet D. J. Brubaker M. A. (2017). cryoSPARC: algorithms for rapid unsupervised cryo-EM structure determination. Nat. Methods 14, 290–296. doi: 10.1038/nmeth.4169 28165473

[B70] Rawson S. Bisson C. Hurdiss D. L. Fazal A. Mcphillie M. J. Sedelnikova S. E.. (2018). Elucidating the structural basis for differing enzyme inhibitor potency by cryo-EM. PNAS 115, 1795–1800. doi: 10.1073/pnas.1708839115 29434040 PMC5828572

[B71] Reyes-Prieto A. Moustafa A. (2012). Plastid-localized amino acid biosynthetic pathways of Plantae are predominantly composed of non-cyanobacterial enzymes. Sci. Rep. 2, 955. doi: 10.1038/srep00955 23233874 PMC3518814

[B72] Rohou A. Grigorieff N. (2015). CTFFIND4: Fast and accurate defocus estimation from electron micrographs. J. Struct. Biol. 192, 216–221. doi: 10.1016/j.jsb.2015.08.008 26278980 PMC6760662

[B73] Ruszkowski M. (2018). Guarding the gateway to histidine biosynthesis in plants: Medicago truncatula ATP-phosphoribosyltransferase in relaxed and tense states. Biochem. J. 475, 2681–2697. doi: 10.1042/BCJ20180289 30072492

[B74] Ruszkowski M. Dauter Z. (2016). Structural studies of medicago truncatula histidinol phosphate phosphatase from inositol monophosphatase superfamily reveal details of penultimate step of histidine biosynthesis in plants. J. Biol. Chem. 291, 9960–9973. doi: 10.1074/jbc.M115.708727 26994138 PMC4859000

[B75] Ruszkowski M. Dauter Z. (2017). Structures of medicago truncatula L-histidinol dehydrogenase show rearrangements required for NAD(+) binding and the cofactor positioned to accept a hydride. Sci. Rep. 7, 10476. doi: 10.1038/s41598-017-10859-0 28874718 PMC5585171

[B76] Rutkiewicz M. Nogues I. Witek W. Angelaccio S. Contestabile R. Ruszkowski M. (2023). Insights into the substrate specificity, structure, and dynamics of plant histidinol-phosphate aminotransferase (HISN6). Plant Physiol. Biochem. 196, 759–773. doi: 10.1016/j.plaphy.2023.02.017 36842242

[B77] Saika H. Fruh T. Iwasaki G. Koizumi S. Mori I. Hayakawa K. (1993). Synthesis of (2r,3r)-, (2s,3s)-, (2r,3s)- and (2s,3r)-imidazole glycerol phosphates (Igp) - substrates for igp-dehydratase (Igpd). Bioorganic Medicinal Chem. Lett. 3, 2129–2134. doi: 10.1016/S0960-894X(01)81031-3

[B78] Saitou N. Nei M. (1987). The neighbor-joining method - a new method for reconstructing phylogenetic trees. Mol. Biol. Evol. 4, 406–425. doi: 10.1093/oxfordjournals.molbev.a040454 3447015

[B79] Sakiroglu M. Ilhan D. (2021). *Medicago sativa* species complex: Revisiting the century-old problem in the light of molecular tools. Crop Sci. 61, 827–838. doi: 10.1002/csc2.20316

[B80] Scheres S. H. (2012). RELION: implementation of a Bayesian approach to cryo-EM structure determination. J. Struct. Biol. 180, 519–530. doi: 10.1016/j.jsb.2012.09.006 23000701 PMC3690530

[B81] Seligmann H. (2003). Cost-minimization of amino acid usage. J. Mol. Evol. 56, 151–161. doi: 10.1007/s00239-002-2388-z 12574861

[B82] Shannon P. Markiel A. Ozier O. Baliga N. S. Wang J. T. Ramage D.. (2003). Cytoscape: A software environment for integrated models of biomolecular interaction networks. Genome Res. 13, 2498–2504. doi: 10.1101/gr.1239303 14597658 PMC403769

[B83] Sinha S. C. Chaudhuri B. N. Burgner J. W. Yakovleva G. Davisson V. J. Smith J. L. (2004). Crystal structure of imidazole glycerol-phosphate dehydratase - Duplication of an unusual fold. J. Biol. Chem. 279, 15491–15498. doi: 10.1074/jbc.M312733200 14724278

[B84] Smith P. M. Atkins C. A. (2002). Purine biosynthesis. Big in cell division, even bigger in nitrogen assimilation. Plant Physiol. 128, 793–802. doi: 10.1104/pp.010912 11891236 PMC1540216

[B85] Stepansky A. Leustek T. (2006). Histidine biosynthesis in plants. Amino Acids 30, 127–142. doi: 10.1007/s00726-005-0247-0 16547652

[B86] Sterling T. Irwin J. J. (2015). ZINC 15–Ligand discovery for everyone. J. Chem. Inf Model. 55, 2324–2337. doi: 10.1021/acs.jcim.5b00559 26479676 PMC4658288

[B87] Tada S. Hatano M. Nakayama Y. Volrath S. Guyer D. Ward E.. (1995). Insect-cell expression of recombinant imidazoleglycerolphosphate dehydratase of arabidopsis and wheat and inhibition by triazole herbicides. Plant Physiol. 109, 153–159. doi: 10.1104/pp.109.1.153 7480319 PMC157571

[B88] Terwilliger T. C. Grosse-Kunstleve R. W. Afonine P. V. Moriarty N. W. Zwart P. H. Hung L. W.. (2008). Iterative model building, structure refinement and density modification with the PHENIX AutoBuild wizard. Acta Cryst. D 64, 61–69. doi: 10.1107/S090744490705024X 18094468 PMC2394820

[B89] Trott O. Olson A. J. (2010). AutoDock Vina: improving the speed and accuracy of docking with a new scoring function, efficient optimization, and multithreading. J. Comput. Chem. 31, 455–461. doi: 10.1002/jcc.21334 19499576 PMC3041641

[B90] Tyohemba R. L. Humphries M. S. Schleyer M. H. Porter S. N. (2022). Accumulation of commonly used agricultural herbicides in coral reef organisms from iSimangaliso Wetland Park, South Africa*. Environ. pollut. 294, 118665. doi: 10.1016/j.envpol.2021.118665 34902525

[B91] Vaughan T. G. (2017). IcyTree: rapid browser-based visualization for phylogenetic trees and networks. Bioinformatics 33, 2392–2394. doi: 10.1093/bioinformatics/btx155 28407035 PMC5860111

[B92] Vázquez D. E. Balbuena M. S. Chaves F. Gora J. Menzel R. Farina W. M. (2020). Sleep in honey bees is affected by the herbicide glyphosate. Sci. Rep. 10, 10516. doi: 10.1038/s41598-020-67477-6 32601296 PMC7324403

[B93] Wang L. Liu R. Y. Li F. Meng Y. Lu H. Z. (2021b). Unveiling the novel characteristics of IGPD polymer and inhibitors binding affinities using 12-6-4 LJ-type nonbonded Mn2+ model. J. Mol. Liq 322, 114992. doi: 10.1016/j.molliq.2020.114992

[B94] Wang L. Liu R. Meng Y. Li F. Lu H. (2021a). Structure and function of the refined C-terminal loop in imidazole glycerol phosphate dehydratase from different homologs. J. Agric. Food Chem. 69, 13871–13880. doi: 10.1021/acs.jafc.1c04282 34780187

[B95] Wang Y. Wang G. Moitessier N. Mittermaier A. K. (2020). Enzyme kinetics by isothermal titration calorimetry: Allostery, inhibition, and dynamics. Front. Mol. Biosci. 7. doi: 10.3389/fmolb.2020.583826 PMC7604385 33195429

[B96] Wiater A. Hulanicka D. Klopotowski T. (1971a). Structural requirements for inhibition of yeast imidazoleglycerol phosphate dehydratase by triazole and anion inhibitors. Acta Biochim. Pol. 18, 289–297. 4942936

[B97] Wiater A. Klopotowski T. Bagdasarian G. (1971b). Synergistic inhibition of plant imidazoleglycerol phosphate dehydratase by aminotriazole and phosphate. Acta Biochim. Pol. 18, 309–314. 4942937

[B98] Wiater A. Krajewska-Grynkiewicz K. Klopotowski T. (1971c). Histidine biosynthesis and its regulation in higher plants. Acta Biochim. Pol. 18, 299–307. 4331571

[B99] Witek W. Sliwiak J. Ruszkowski M. (2021). Structural and mechanistic insights into the bifunctional HISN2 enzyme catalyzing the second and third steps of histidine biosynthesis in plants. Sci. Rep. 11, 9647. doi: 10.1038/s41598-021-88920-2 33958623 PMC8102479

[B100] Witte C. P. Herde M. (2020). Nucleotide metabolism in plants. Plant Physiol. 182, 63–78. doi: 10.1104/pp.19.00955 31641078 PMC6945853

[B101] Wolter F. P. Fritz C. C. Willmitzer L. Schell J. Schreier P. H. (1988). Rbcs genes in solanum-tuberosum - conservation of transit peptide and exon shuffling during evolution. PNAS 85, 846–850. doi: 10.1073/pnas.85.3.846 3422467 PMC279652

[B102] Wood C. Burnley T. Patwardhan A. Scheres S. Topf M. Roseman A.. (2015). Collaborative computational project for electron cryo-microscopy. Acta Cryst. D 71, 123–126. doi: 10.1107/S1399004714018070 25615866 PMC4304692

[B103] Young N. D. Debellé F. Oldroyd G. E. D. Geurts R. Cannon S. B. Udvardi M. K.. (2011). The *Medicago* genome provides insight into the evolution of rhizobial symbioses. Nature 480, 520–524. doi: 10.1038/nature10625 22089132 PMC3272368

[B104] Zallot R. Oberg N. Gerlt J. A. (2019). The EFI web resource for genomic enzymology tools: Leveraging protein, genome, and metagenome databases to discover novel enzymes and metabolic pathways. Biochemistry 58, 4169–4182. doi: 10.1021/acs.biochem.9b00735 31553576 PMC7057060

[B105] Zerbe B. S. Hall D. R. Vajda S. Whitty A. Kozakov D. (2012). Relationship between hot spot residues and ligand binding hot spots in protein-protein interfaces. J. Chem. Inf Model. 52, 2236–2244. doi: 10.1021/ci300175u 22770357 PMC3623692

