# Supplementary material for: Targeting imidazole-glycerol phosphate dehydratase in plants: novel approach for structural and functional studies, and inhibitor blueprinting
Source: Front Plant Sci. 2024 Mar 15;15:1343980. doi: 10.3389/fpls.2024.1343980 (PMC10978614; doi:10.3389/fpls.2024.1343980)
Supplement: Supplementary file 1 [file DataSheet_1.pdf]

SUPPLEMENTARY MATERIAL

Targeting imidazole-glycerol phosphate dehydratase in plants: novel approach for structural and functional studies, and inhibitor blueprinting

Wojciech Witek[1], Joanna Sliwiak[1], Michal Rawski[2], Milosz Ruszkowski[1*]

[1]Department of Structural Biology of Eukaryotes, Institute of Bioorganic Chemistry, Polish Academy of Sciences, Poznan, Poland

[2]SOLARIS National Synchrotron Radiation Centre, Krakow, Poland

*Correspondence:
Milosz Ruszkowski
Department of Structural Biology of Eukaryotes,
Institute of Bioorganic Chemistry,
Polish Academy of Sciences,
Noskowskiego 12/14,
Poznan 61-704, Poland
E-mail: mruszkowski@ibch.poznan.pl

Supplementary Figure S1. Electron density maps
Supplementary Figure S2. Phylogenetic tree.
Supplementary Figure S3. Highest-scoring results of the virtual screening in the cleft between two active sites.
Supplementary Figure S4. Spectral absorption plots for imidazole-glycerol phosphate (IGP) enzymatic synthesis

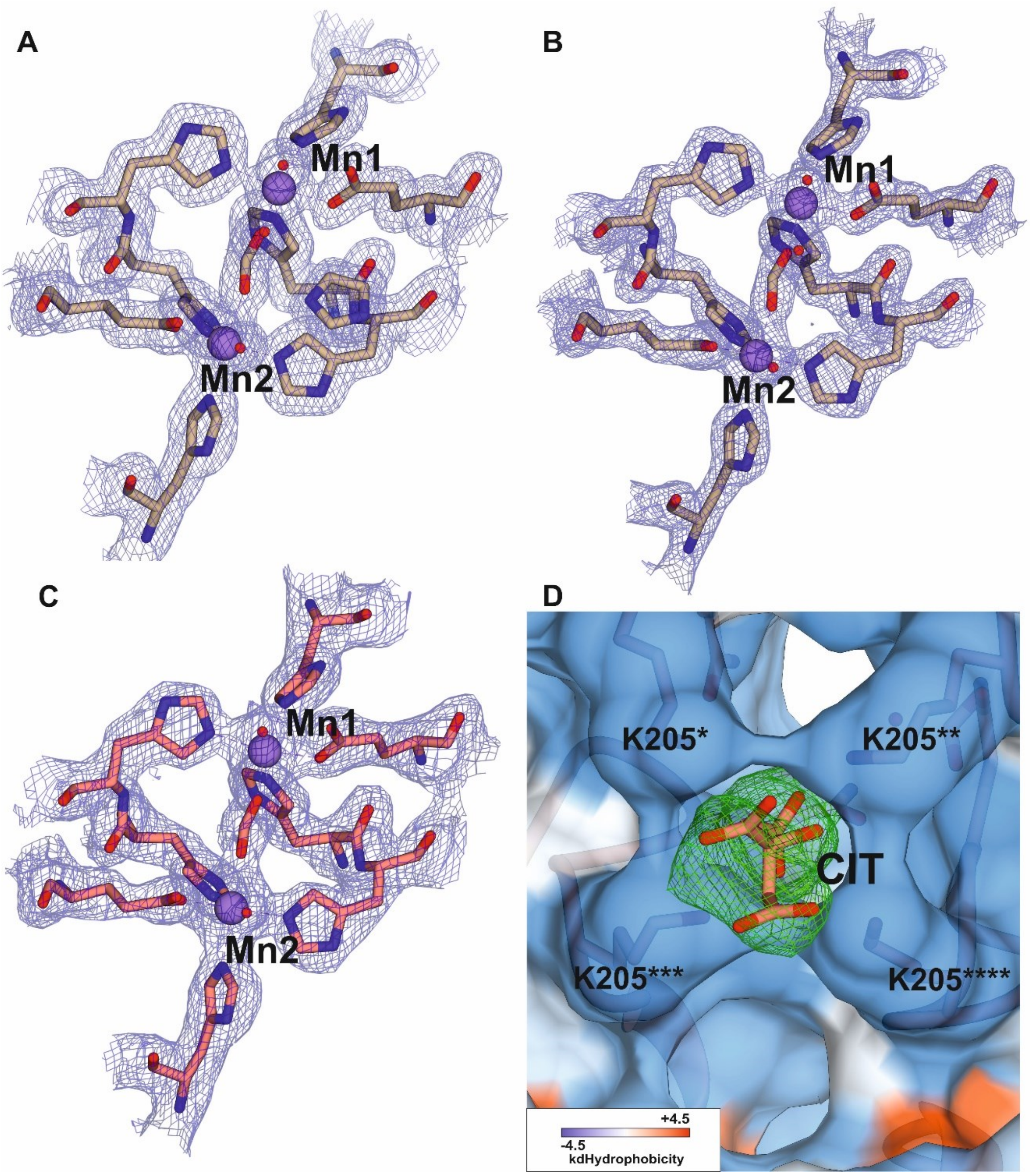

Supplementary Figure S1.
Electron density maps. Panels A-C show $2F_o$-F_c electron density maps around active sites in crystal structures of *Mt*HISN5: 1.55 Å, PDB ID: 8QAW (σ = 1.0); 1.69 Å, 8QAX (σ = 1.7); and 2.20 Å, 8QAY (σ = 1.0), respectively. Panel D represents a polder map around a citrate molecule bound in 8QAY (σ = 4.6). Citrate is bound at the tunnel along a non-crystallographic four-fold symmetry axis and is stabilized by side chains of Lys205 and its counterparts from other chains which are indicated by asterisks. *Mt*HISN5 surface is 20% transparent and coloring is based on hydrophobicity.

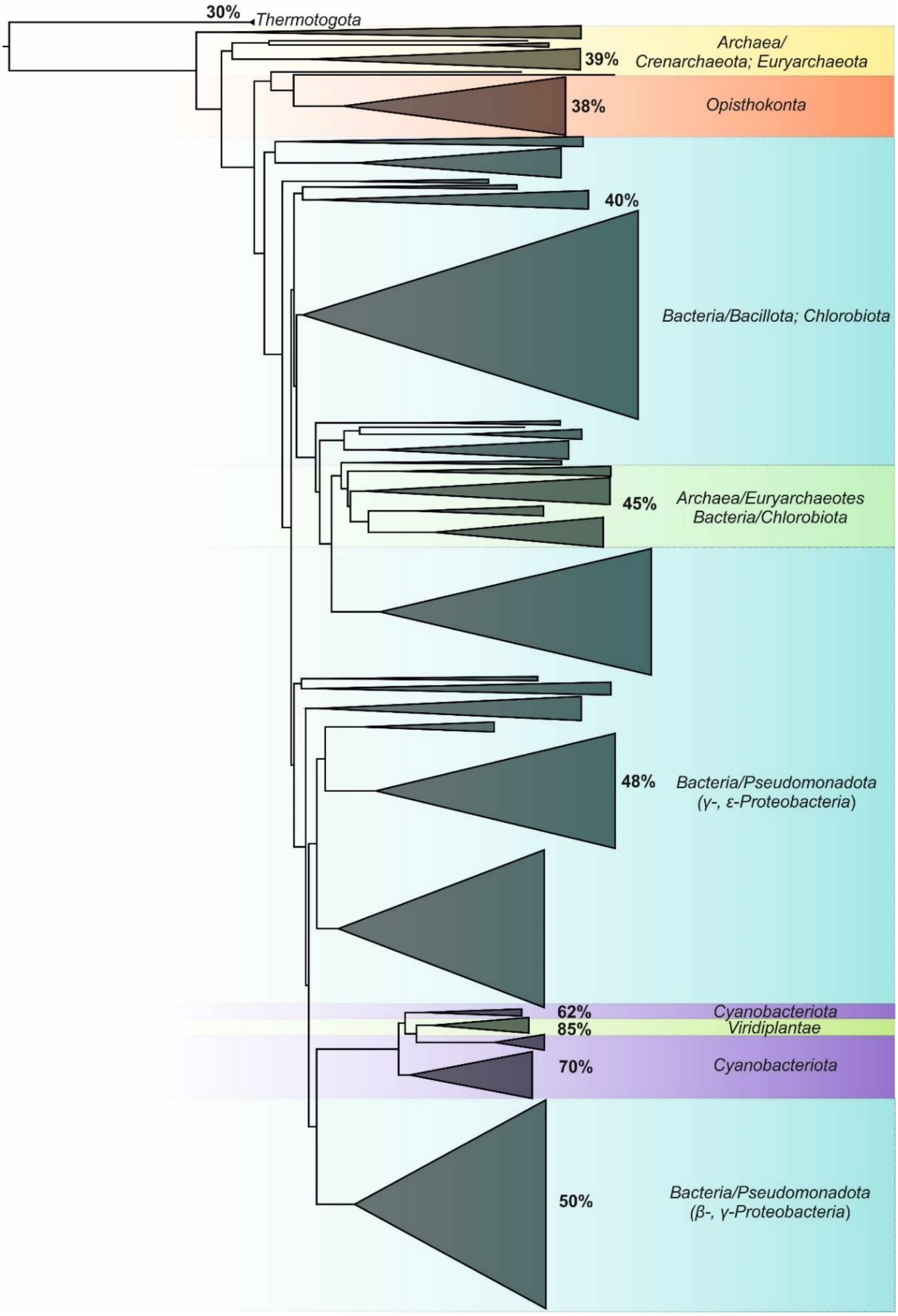

Supplementary Figure S2.
Outgroup rooted (*Termotogota*) phylogenetic tree of *Mt*HISN5 homologs. The tree shows close relationship of cyanobacterial and plant (*Viridiplantae*) sequences. Other eukaryotic sequences, i.e. *Opisthokonta*, are closer to *Archaea* and *Bacillota/Chlorobiota.* Total number of 478 protein sequences were analyzed and grouped to enhance data clarity. Percent values indicate amino acid sequence identity to the sequence of *Mt*HISN5.

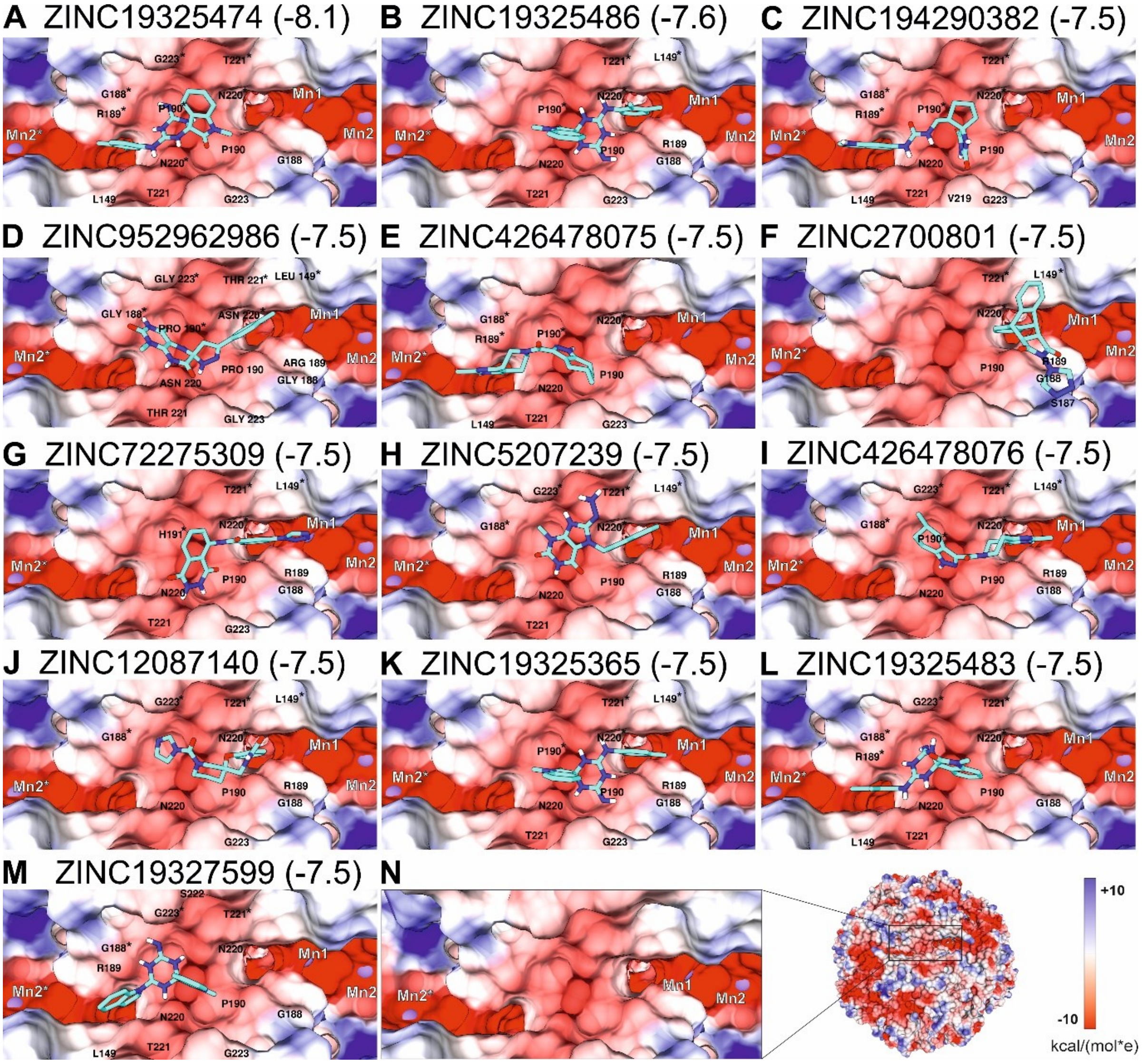

Supplementary Figure S3.

Highest-scoring results of the virtual screening calculated for the cleft between two active sites of adjacent *Mt*HISN5 subunits. (A-M) ZINC IDs are given together with the estimated energy gain (in parentheses, kcal/mol). (N) Fragment of the *Mt*HISN5 surface. *Mt*HISN5 surface is colored by coulombic potential according to the color key (bottom-right corner). The active sites are marked by Mn1, Mn2 and Mn2* from the adjacent subunit.

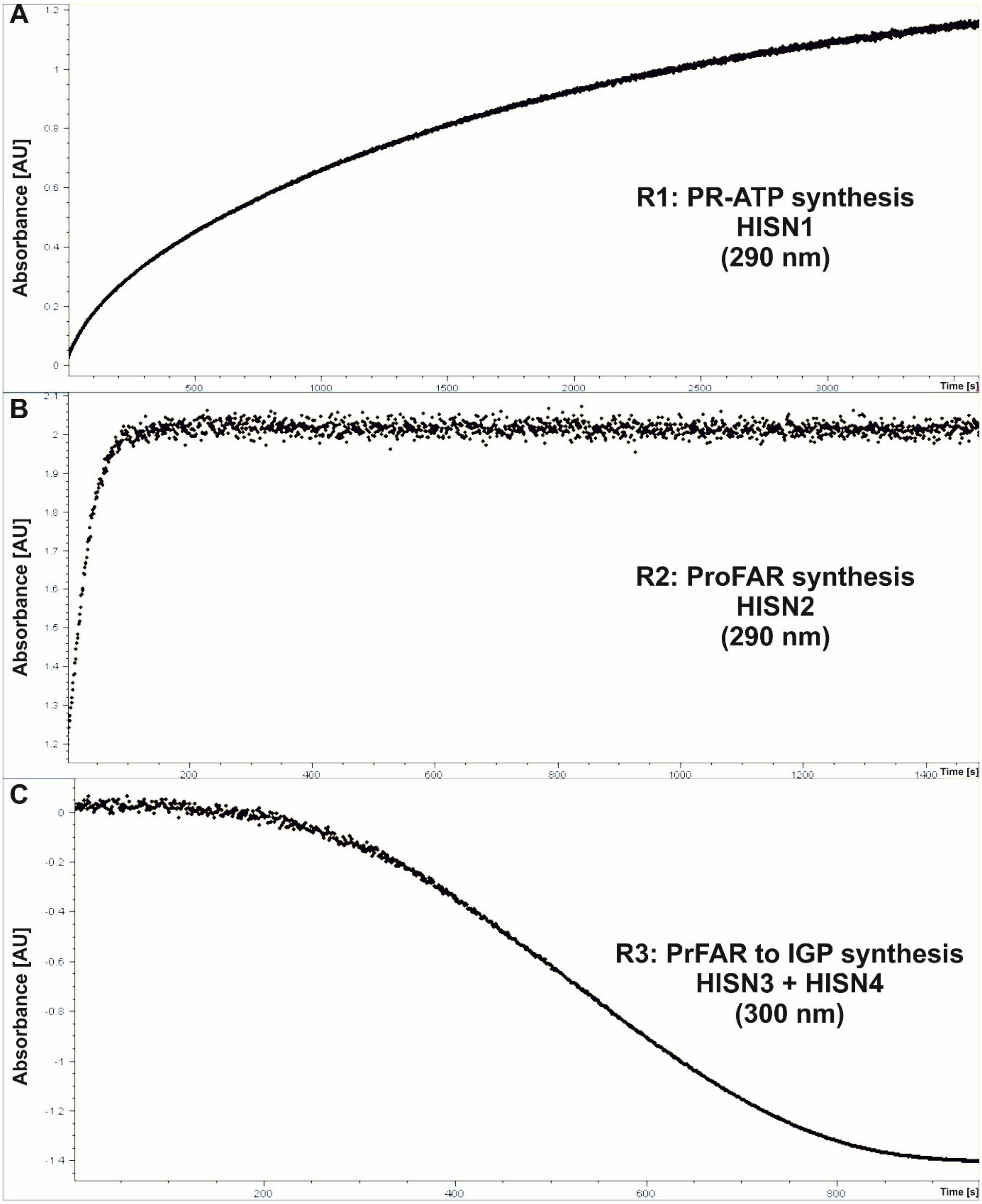

Supplementary Figure S4. Absorption spectra for IGP synthesis. Panel A shows absorption increase at 290 nm owed to production of PR-ATP. Panel B represents PR-ATP conversion to ProFAR, monitored at 290 nm. Panel C shows ProFAR isomerization to PrFAR by HISN3 and immediate PrFAR conversion to IGP, which starts as an absorption decrease at 300 nm after approx. 150 sec.
